# Sentiment analysis techniques, challenges, and opportunities: Urdu language-based analytical study

**DOI:** 10.7717/peerj-cs.1032

**Published:** 2022-08-31

**Authors:** Muhammad Irzam Liaqat, Muhammad Awais Hassan, Muhammad Shoaib, Syed Khaldoon Khurshid, Mohamed A. Shamseldin

**Affiliations:** 1 Department of Computer Science, University of Engineering and Technology Lahore, Lahore, Punjab, Pakistan; 2 Dept. of Mechanical Engineering, Faculty of Engineering Technology, Future University in Egypt, New Cairo, Eygpt

**Keywords:** Sentiment analysis, Opinion mining, Poor resource language, Word sensedisambiguation, Urdu-based language constructs, Digital repositories

## Abstract

Sentiment analysis in research involves the processing and analysis of sentiments from textual data. The sentiment analysis for high resource languages such as English and French has been carried out effectively in the past. However, its applications are comparatively few for resource-poor languages due to a lack of textual resources. This systematic literature explores different aspects of Urdu-based sentiment analysis, a classic case of poor resource language. While Urdu is a South Asian language understood by one hundred and sixty-nine million people across the planet. There are various shortcomings in the literature, including limitation of large corpora, language parsers, and lack of pre-trained machine learning models that result in poor performance. This article has analyzed and evaluated studies addressing machine learning-based Urdu sentiment analysis. After searching and filtering, forty articles have been inspected. Research objectives have been proposed that lead to research questions. Our searches were organized in digital repositories after selecting and screening relevant studies. Data was extracted from these studies. Our work on the existing literature reflects that sentiment classification performance can be improved by overcoming the challenges such as word sense disambiguation and massive datasets. Furthermore, Urdu-based language constructs, including language parsers and emoticons, context-level sentiment analysis techniques, pre-processing methods, and lexical resources, can also be improved.

## Introduction

Sentiment analysis (SA), otherwise known as opinion mining, is the field of research that involves processing and analyzing opinions, behaviors, and the sentiments of the people towards specific issues, events, organizations, products, services, or their respective attributes (Liu, 2012). These sentiments are often retrieved from the big textual data available to us through social media and the world wide web. Moreover, social media and its rapid growth in the world have encouraged a whole new dimension of knowledge that contains a vast range of sentiment over events, incidents, issues, or any modern-day dilemmas (Asghar et al., 2017). Nowadays, sentiment analysis is considered crucial in every field and, therefore, plays a critical role in the decision-making process across organizations, industries, communities, and even governments. With the advancements in natural language processing (NLP) with machine learning and deep learning algorithms, the applications of sentiment analysis are increasing at an immense rate. Although, for languages like English and French, these applications have delivered great performance, however, these applications are approaching maturity for Urdu-based sentiment analysis (Khan et al., 2021a). Customer behavior analysis, public response, user reviews, and trends on social media platforms are among the key areas that are being explored by using the Urdu-based sentiment analysis. The English language, for instance, has been able to establish a vast and diverse collection of resources, including many parsers, lexicons, taggers for parts of speech, and a wide range of NLP tools (Anwar, Wang & Wang, 2006), thus, encouraging and strengthening the robustness of the decision-making process across various fields of life. However, simultaneously, SA lacks resource-deprived languages (such as Urdu) due to the non-availability of the resources as mentioned earlier for these languages. Additionally, while most of the SA systems are designed and developed to be in resource-rich languages, the analysis of big data from social media and other platforms in poor resource languages poses a challenge to the researchers (Dashtipour et al., 2016). Meanwhile, Urdu is the national language of Pakistan, which is widely spoken and understood by millions of people worldwide; there is a need to construct and maintain high sentiment and lexical resources for this language. Furthermore, despite having several publications for the task of SA in Roman Urdu language, the literature lacks in terms of presenting a vast range of resources such as parsers, lexicons, and corpora as compared to those of resource-rich languages such as English and French (Naqvi, Majid & Abbas, 2021). While the resources from other languages such as English and French are available in sufficient quantity however, using these for Urdu SA results in poor performance mainly due to complex sentence structure, and word sense disambiguation. Awais & Shoaib (2019) investigated the use of several techniques used for sentiment analysis for other languages including English and reported that these techniques fail to replicate the same results for Urdu. The Urdu text is mainly categorized into two major categories including Arabic Script and the Roman Urdu Scripts. The literature suggests that the underlying problem areas remain the same for both kinds of script due to the linguistic constraints of the language. However, flexibility in the language with words from other languages, morphological variability, case markers, and emoticons are few of the distinctive peculiarities of Urdu language that are associated with the Arabic script. Apart from these factors, Roman Urdu script of the language has respective challenges associated with it for performing effective sentiment analysis. The key challenges for the roman Urdu-based SA include slangs stockpiles, negations and modifiers, sentiment lexicons, and scarce datasets (Syed, Aslam & Martinez-Enriquez, 2010). Additionally, a recent study outlined the need for a comprehensive study aimed at exploring the machine and deep learning-based approaches for Urdu SA (Khattak et al., 2021). These surveys highlighted that apart from the resource-related challenges for Urdu SA, at the same time, secondary studies focusing on the machine learning-based Urdu SA are very few (Anwar, Wang & Wang, 2006; Khattak et al., 2021; Khan et al., 2019; Lo et al., 2017). Therefore, presenting an opportunity to synthesize the overall existing research addressing ML and DL approaches used for Urdu-based sentiment analysis. This study is aimed at highlighting the research dimensions that were missed in the previously existing studies, including quality assessment scoring, publication channels, ML and DL approaches, the effectiveness of ML techniques, encountered challenges, and future opportunities. Moreover, this systematic literature review (SLR) outlines these research dimensions by providing a comprehensive analysis of studies addressing ML and DL-assisted approaches adopted for Urdu-based SA. Additionally, this study aims to deliver the encountered challenges and possible future directions in machine learning-based Urdu sentiment analysis. To this effect, along with the quality assessment criteria, we have defined detailed inclusion and exclusion criteria for selecting and rejecting the studies. Based on the specified criteria for the systematic review, we have shortlisted 40 articles for detailed analysis. In this way, we have only considered the studies published in impact factor journals or top-tier conferences and excluded all the studies that failed on the quality assessment criteria. In a nutshell, this systematic literature review’s novelty includes identifying the relevant publication channels for Urdu-based SA, classification of existing ML-based studies, and identification of the opportunities and challenges for this task. Additionally, the study shall assist the researchers by presenting the existing research within a single umbrella to understand the Urdu-based SA better. The remaining article is structured as follows: Section II outlines the literature review and the motivation. Research methodology, research questions, and research objectives are discussed in Section III. The results of these questions are evaluated and analyzed in Section IV of the article. Section V explains the taxonomy of the carried out systematic literature review in light of the explored results. Finally, the study is concluded in Section VI.

## Literature Review

In recent years, sentiment analysis has evolved greatly as a domain in Natural language processing. With the rapid growth of social media and world wide web, the importance of extracting meaning from the big data has observed a great hike. The manual analysis of this textual big data generated by the web is costly and therefore, intelligent solutions such as sentiment analysis or opinion mining are in great demand. These methods are used in variety of fields ranging from opinion mining for different products in marketing to observing public response towards governmental policies. While existing studies aim to explore the domain with rich resource languages such as English and French, this systematic literature will present the existing research in context of Urdu-based sentiment analysis. Moreover, it will serve as the baseline for the interested researchers and technologist aiming to explore the recent trends, challenges, and opportunities in the field of Urdu-based sentiment analysis. Sentiment analysis for Urdu corpora is developing to achieve maturity for performing robust Urdu-based SA. Compared to other high resource languages such as English, the field lacks comprehensive studies synthesizing the existing work carried out in this research domain. Most of the existing studies lack classifying the works based on the publication channels and machine learning approaches. They tend to focus on the development of sentiment lexicon, pre-processing techniques, and the role of modifiers. Table 1 presents a more recent survey (Khattak et al., 2021) on the sentiment analysis for the Urdu language addressed three different dimensions of Urdu-based SA, including pre-processing, lexicon resources, and sentiment classification. This research remains the only study in this domain area that has followed and presented the systematic approach of SLR and listed their results, according to our best knowledge. While it is the most recent article on the issue that focuses on the Urdu-based SA, it also has highlighted that the literature lacks classification and evaluation of machine and deep learning approaches for Urdu-based SA. However, the study’s shortcoming includes not discussing the earlier defined issues for the machine-learning-based Urdu SA approaches. Another study presented by Khan et al. (2019) reviewed 14 articles published in the domain of Urdu-based SA. The authors classified the studies using lexicon-based, machine learning, and hybrid approaches. However, the shortcoming of the study includes that while it is not a recent study and more work has been performed in the research area, at the same time, it lacks the comprehensiveness that can be used to generalize its findings to the overall research domain. On the other hand, different methods of lexicon development and evaluation are evaluated by Anwar, Wang & Wang (2006) that involved the studies employing the use of part of speech tagging (POS), name entity recognition (NER), and parsing. However, the study lacked in identifying all the techniques specifically adopted for resource-deprived languages that the study aimed to address primarily. Another study by Lo et al. (2017) addressed the issue of resource-deprived languages for multilingual sentiment analysis. The study emphasized investigating and evaluating different tools and techniques for this purpose and listed the challenges and future recommendations. However, our work is superior to these works as it focuses on the classification of different machine and deep learning-based approaches that the researchers in the domain area have employed. Additionally, it distinguishes itself from the rest of the studies as it attempts to present the complete research process through a systematic literature survey, defining the strict inclusion criteria and systematic evaluation of the posed research questions.

**Table 1:** Comparison with related work.

Article	Focus of the study	Ref.	Approach	Quality assessment score	Identification of dimensions				Explored survey perspectives	Targeted repositories
						Publication channels	Machine learning approaches	Challenge and opportunities	Effective machine learning methods	
Khattak et al. (2021)	Survey of sentiment analysis in Urdu language	2020	SLR	Yes	Yes	Yes	No	Yes	No	3
Khan et al. (2019)	Survey of the lexicon, machine learning, and hybrid techniques for SA	2019	Trad.	No	No	No	Yes	No	No	Not mentioned
Anwar, Wang & Wang (2006)	Review of studies for lexicon development	2006	Trad.	No	Yes	No	No	Yes	No	Not mentioned
Lo et al. (2017)	Survey of multilingual based SA	2017	Trad.	No	Yes	No	No	Yes	No	Not mentioned
This survey	Classification of machine and deep learning approaches	2021	SLR	Yes	Yes	Yes	Yes	Yes	Yes	10

## Research Methodology

We have followed the systematic literature review approach that is proposed by Brereton et al. (2007) and Kitchenham (2004). The guidelines from these studies are considered for finalizing the research question and thus, eliminating any biases from the research process.

The adopted protocol comprised three stages: planning the review, conducting the study, and reporting the review’s findings. These stages have been explained in detail in the coming sections.

### Review plan

**Figure 1:**
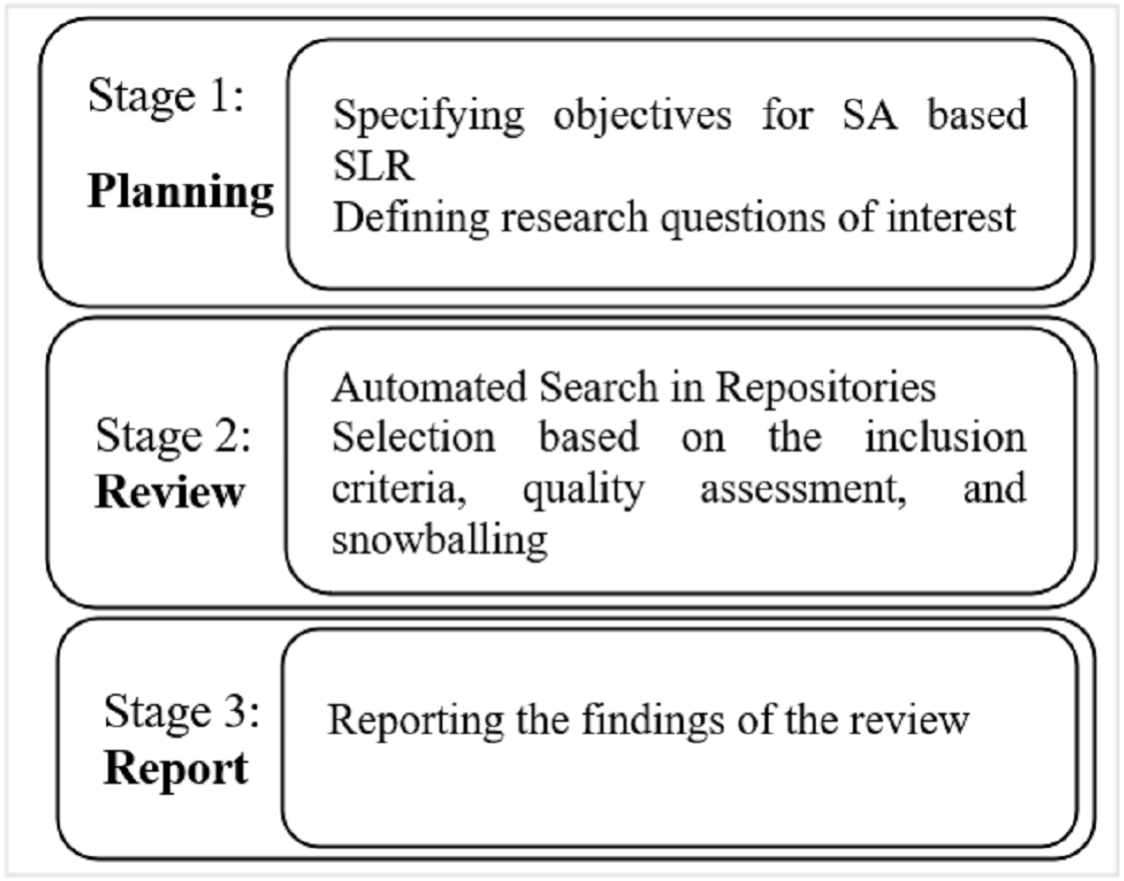
Research methodology.

**Figure 2:**
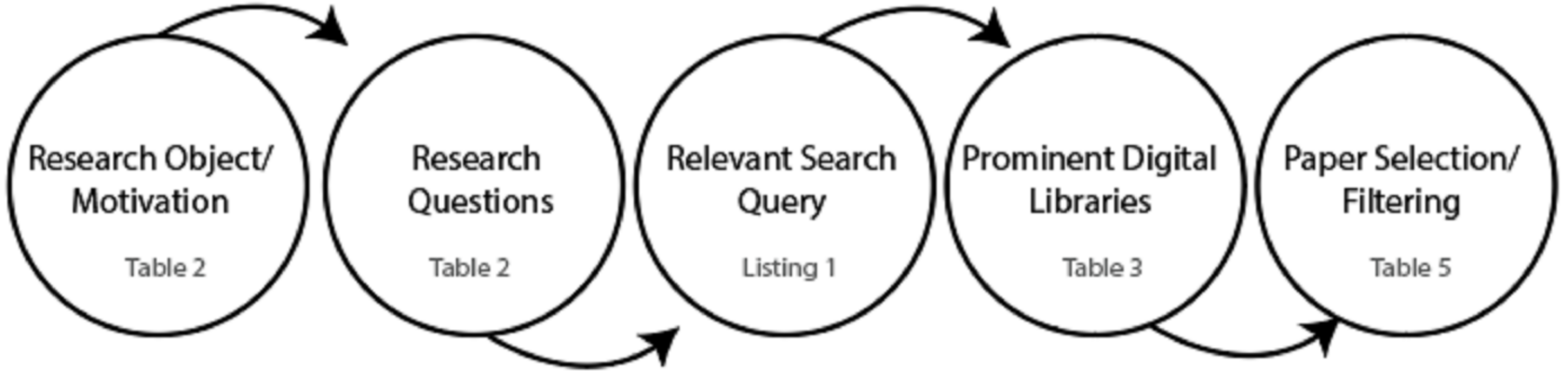
Search strategy.

We have defined a systematic approach to extract the relevant studies from the literature. Moreover, a pictorial depiction of the research strategy is visualized in Figs. 1 and 2 that outlines the research strategy. The formal steps included for this purpose include:

• Research objectives
• Organized searches in digital repositories
• Studies selection
• Screening of relevant studies
• Data extraction from these studies
• Synthesizing the results
• Writing the review report

The study by Brereton et al. (2007) and Kitchenham (2004) requires the formulation of a research question for achieving the research objectives. Therefore, these research questions have been formulated and presented in Table 2.

**Table 2:** Research questions.

**RQ**	**Research question statement**	**Objectives and motivation**
RQ1:	What are the relevant publication channels for Urdu-based SA?	To identify 1. High quality publication channels 2. research carried out during Jan 2012-Nov 2021
RQ2:	What are the primary studies that have studied and discussed the use of machine learning methods for sentiment analysis?	To identify 1. Different machine learning-based techniques used for SA by researchers across the world 2. Impact of such techniques on overall performance 3. Recent trends in SA
RQ3:	What are the major challenges and opportunities for the Urdu-based sentiment analysis?	To identify 1. Challenges that the researchers have faced throughout these past years 2. Future directions for the researchers to facilitate the research in this domain area
RQ4:	What are the most effective machine learning-based methods for performing Urdu-based SA?	To identify 1. Machine learning-based methods that the researchers use to perform the Urdu-based SA in the literature 2. Which methods delivered better performance results based on the F1-score, accuracy, precision metrics

• The RQ1 aims to explore and identify different high-value repositories that have published literature on the problem of Urdu-based sentiment analysis. Answering this question shall allow us to pinpoint the venues that can be prioritized for credibility and scoring for better literature evaluation.
• Another objective of this is to identify the primary studies that have been conducted in the span of the last four years, focusing on the issues of machine learning-based sentiment analysis. The RQ2 shall assist in the identification and evaluation of these studies.
• The objective of finding the challenges and opportunities in Urdu-based SA shall be addressed through RQ3.
• Finally, the most effective machine learning methods shall be identified throughout the selected published literature that has delivered an excellent performance for SA. The assessment of this research objective shall be carried out by evaluating the RQ4.

### Review conduct

We have conducted this systematic literature review using the four below-mentioned steps. Initially, we have extracted the most relevant studies from the selected digital repositories. Further, the pre-defined inclusion and exclusion criteria are used to either include or exclude the reflections in the survey. Moreover, designing the quality assessment criteria greatly enhanced the effectiveness of our approach, which is described in the third step. Finally, the most important articles are backtracked by adopting the snowballing approach in the last stage.

#### Automated search in digital repositories

To filter out the irrelevant studies and ensure the robustness of the research process, we have designed and employed automated and manual searching techniques to extract useful information from the most relevant studies. Moreover, globally accepted digital libraries, also considered in previous similar surveys, are selected to perform the systematic literature review. In addition, we have considered google scholar, a globally accepted engine that retrieves the best literature by using various filters. Furthermore, these digital libraries for automated research include:

• IEEE eXplore (https://ieeexplore.ieee.org/)
• SpringerLink (https://link.springer.com/)
• ACM Digital Library (https://dl.acm.org/)
• PLOS ONE (https://journals.plos.org/plosone/)
• arXiv (https://arxiv.org/search/cs)
• IGI Global (https://www.igi-global.com/search/)
• Wiley Library (https://onlinelibrary.wiley.com/)
• European Library (https://www.ceeol.com/)
• Central and Eastern European Online Library (https://www.ceeol.com/)
• Google Scholar (https://scholar.google.com/)

The query string is formulated using the following guidelines.

• Identification of the primary keywords by considering the RQs
• Identification of secondary keywords by using the synonyms and additional keywords for primary keywords
• Incorporating the use of ‘AND’ and ‘OR’ operators between selected primary and secondary keywords
• Wildcard operator ‘*’ is also used with the derived keywords to extract the most suitable and relevant studies.

**Figure 3:**
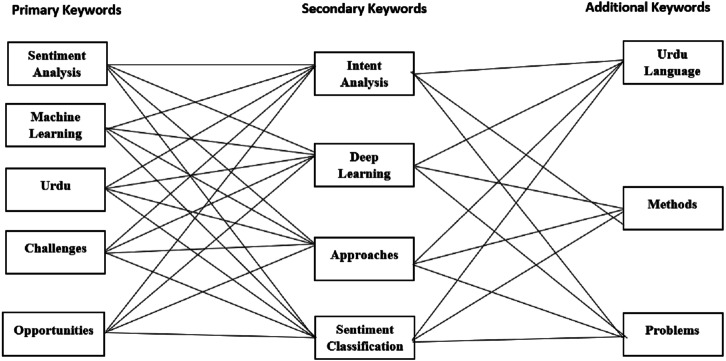
Query string listing possible combinations using derived keywords.

Figure 3 provides the visualization for possible combinations of the query string. Meanwhile, we have listed a sample query string containing a mix of primary and secondary keywords and the Boolean and wildcard operators. The query string is constructed by extracting the primary keywords from the defined research questions for the SLR. Additionally, the alternative keywords are considered using synonyms and alternative keywords for all the primary keywords. Similarly, in this fashion, additional keywords such as “methods,” “language”, and “Urdu language” have been incorporated to facilitate the research process. Finally, the constructed query string retrieves and collects the most relevant articles addressing the Urdu language-based SA. Meanwhile, the query proved to be very effective compared to the traditional approach. However, filtering out studies based on the inclusion criteria became difficult. Table 3 outlines the final versions of the queries used to extract results from the selected digital repositories.

**Table 3:** Scores for journals and conferences.

**Publication source**	**+4**	**+3**	**+2**	**+1**	**+0**
Journals	Q1	Q2	Q3	Q4	No JCR Ranking
Conferences, Workshops, Symposia	CORE A*	CORE A	CORE B	CORE C	Not in CORE Ranking

 
 
        Listing 1: Search Query 
________________________________________________________________________________________________________ 
                                                                                                         ( Sentiment Analysis OR Opinion Mining OR Intent Analysis OR 
                                                                           Sentiment Classification ) AND ( Machine Learning OR Deep Learning 
                                                                           OR Supervised Learning OR Semi - Supervised Learning ) AND ( Urdu 
                                                                           OR Urdu language ) AND ( Challenges ) AND ( Opportunities ) 
___________________________________________________________________________    

This query involved using specific keywords and a set of provided filters by the respective digital libraries. While ACM journals and PLOS ONE were searched with the restriction of title only, other libraries, as they do not offer such configurations, were searched using the “all fields” setting.

#### Selection based on inclusion/exclusion criteria

### A. Inclusion criteria

• Article included must discuss sentiment analysis as the central topic.
• Article must target at least one of the research questions.
• Article must be published in SJR indexed journals.
• Article must be published in top-tier conferences.
• It explores challenges, shortcomings, and various opportunities in Urdu sentiment analysis.
• The study has, in some way, discussed the problems and plausible solutions for opinion mining in resource-poor language.

### Exclusion criteria

• Exclude articles written in non-English.
• Exclude articles not discussing even of the defined RQs.
• Paid articles, student articles, and preprints are also excluded.
• Exclude articles published before 2012.
• Exclude articles written by the same research group repeatedly over time.
• Exclude the previous version of an article with multiple versions (most recent ones were kept only).

#### Selection based on quality assessment

The classification of the study based on the quality is the most subjective and challenging task. The step is considered very important for any review and plays a significant role in the quality of the survey itself. Therefore, we have employed the use of state-of-the-art quality assessment metrics suggested by Fernandez, Insfran & Abrahão (2011) and Ouhbi et al. (2015). Moreover, to strengthen the authenticity of our review, we have designed a questionnaire to assess the quality of each document; additionally, the task is carried out by three different authors of our team. These studies are scored by using the following criteria:

1. The study is assigned score ‘1’ if it discusses the Urdu-based sentiment analysis approaches; otherwise, it is scored ‘0’.
2. Score ‘1’ is awarded to studies discussing machine learning-based sentiment analysis approaches otherwise scored ‘0’.
3. The empirically evaluated studies are similarly awarded a score of ‘1’ else it ‘0’.
4. Studies outlining the challenges and opportunities related to Urdu-based sentiment analysis are assigned a score of ‘1’ otherwise ‘0’.
5. Lastly, the highest weightage is given to the impact factor publications and top tier conferences according to guidelines provided by Portal (2018), and Rank (2018). The scores are listed in Table 3. for different categories.
6. Finally, the total score for each article is calculated by adding all the awarded scores (in the range of zero to eight). The studies with more than four are included for the final review.

#### Selection based on snowballing

The references of the selected articles were also explored to backtrack the essential articles, and several articles are included by adopting the snowballing (Wohlin, 2014) technique. However, only those articles are considered that clear the inclusion criteria and have four or more scores.

### Review report

The next stage of the systematic literature review involves selecting the most relevant articles from the digital repositories. Table 4 outlines the details of the results and filtering that we have performed on the set of ten different digital repositories listed in Table 5. Additionally, we have excluded articles less than four pages and performed the filtering using parameters such as ‘since 2017’, ‘title,’ ‘abstract,’ ‘Introduction and conclusion,’ and lastly, full articles. In this way, the final selected studies were inspected thoroughly and developed a systematic knowledge base for these 40 articles.

**Table 4:** Query string generated results and filtering phases.

**Phase**	**Selection**	**Selection criteria**	**ACM digital library**	**IEEE Xplore**	**PLOS ONE**	**Science direct**	**Springer link**	**Wiley online library**	**arXiv**	**IGI Global**	**CEEOL**	**Google scholar**	**Total articles**
1	Search	Keywords (Fig. 3)	172,796	531	425	14,143	377,221	122	129	1,386	103	50,900	**617,639**
2	Filtering	Since 2017	48,912	492	350	8,903	137,721	89	12	1,386	103	18,500	**216,468**
3	Filtering	Title	170	50	150	128	150	50	6	27	30	198	**820**
4	Filtering	Abstract	103	30	15	40	10	35	3	15	12	58	**303**
5	Filtering	Introduction and conclusion	33	12	3	10	7	15	2	8	5	37	**110**
6	Inspection	Full Article	8	10	1	8	3	5	1	1	2	2	**40**

**Table 5:** Search strategy for selected repositories.

**Digital library**	**Search query**	**Applied filter**
ACM Digital Library	[[All: sentiment analysis] OR [All: intent analysis]] AND [[All: machine learning] OR [All: deep learning]] AND [All: Urdu] AND [All: challenges] AND [All: opportunities]	Since 2017
IEEE Xplore	(Sentiment Analysis OR Sentiment Classification) AND (Machine Learning OR Deep Learning OR Supervised Learning OR Semi Supervised Learning) AND (Urdu OR Urdu language)	Since 2017
PLOS ONE	Urdu Sentiment Analysis using Machine Learning	Since 2017
Google Scholar	(Sentiment Analysis OR Opinion Mining OR Intent Analysis OR Sentiment Classification) AND (Machine Learning OR Deep Learning OR Supervised Learning OR Semi Supervised Learning) AND (Urdu OR Urdu language) AND (Challenges) AND (Opportunities)	Since 2012 Since 2017
Science Direct	(Sentiment Analysis OR Opinion Mining OR Intent Analysis OR Sentiment Classification) AND (Machine Learning OR Deep Learning OR Supervised Learning OR Semi Supervised Learning) AND (Urdu OR Urdu language) AND (Challenges) AND (Opportunities)	Since 2017
Springer Link	(Sentiment Analysis OR Opinion Mining OR Intent Analysis OR Sentiment Classification) AND (Machine Learning OR Deep Learning OR Supervised Learning OR Semi Supervised Learning) AND (Urdu OR Urdu language) AND (Challenges) AND (Opportunities)	Since 2017
Wiley Online Library	(Sentiment Analysis OR Opinion Mining OR Intent Analysis OR Sentiment Classification) AND (Machine Learning OR Deep Learning OR Supervised Learning OR Semi Supervised Learning) AND (Urdu OR Urdu language) AND (Challenges) AND (Opportunities)	Since 2017
arXiv	Urdu Sentiment Analysis using Machine Learning	Since 2017
IGI Global	Expert search-based keywords: Sentiment Analysis, Urdu, Machine Learning, Deep Learning, Challenges, opportunities	Since 2017
CEEOL	Search String keywords: Sentiment Analysis, Urdu, Machine Learning, Deep Learning, Challenges, opportunities	Since 2017

## Assessment and Discussion of Research Questions

The research questions evaluate the insight of selected 40 studies extracted through a systematic literature review.

### Assessment of RQ1: What are the relevant publication channels for Urdu-based SA?

**Figure 4:**
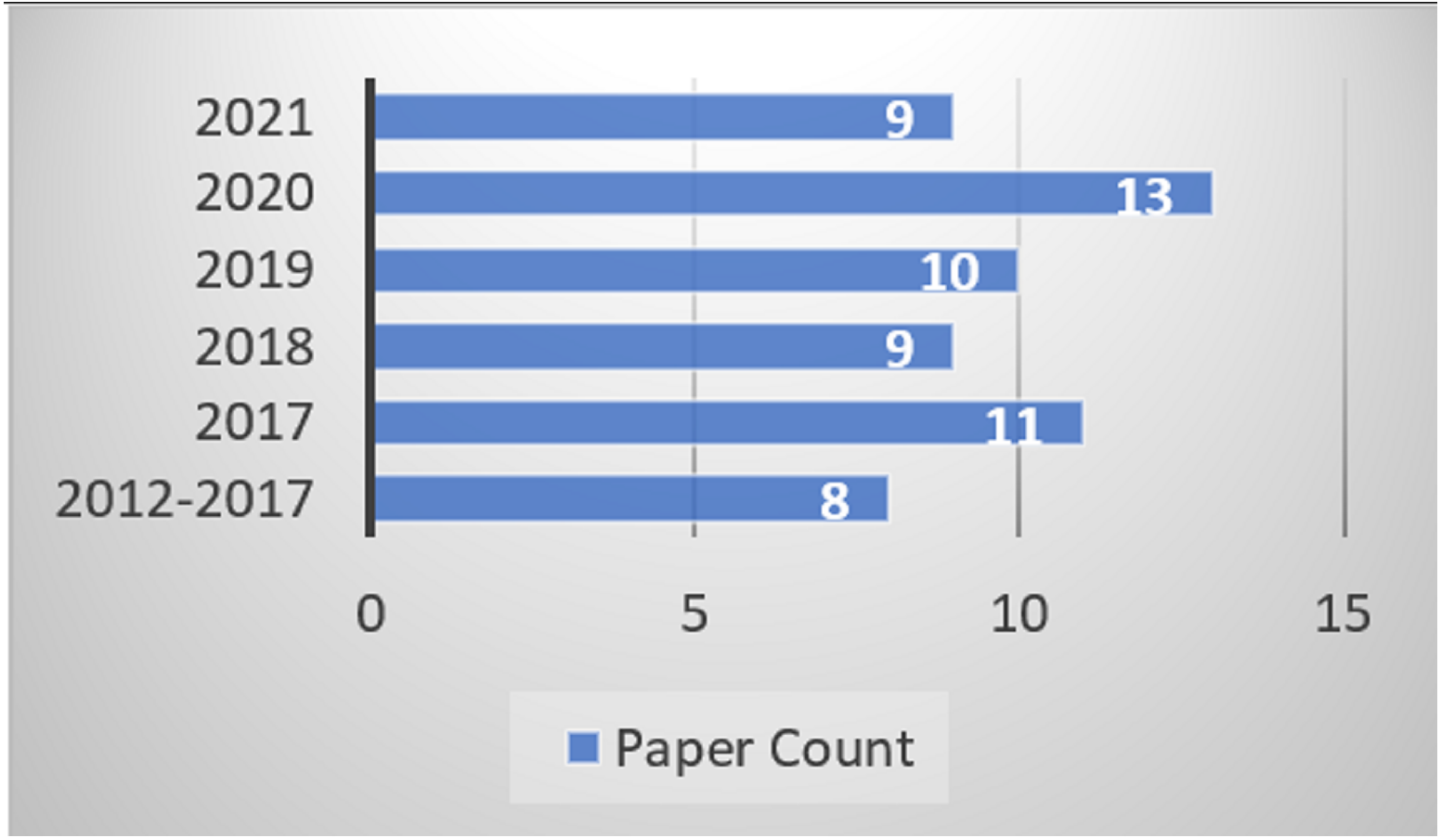
Year wise publication identification.

The Urdu-based Sentiment Analysis remains a challenging field of research for researchers as there are very few resources available for performing the SA. Lack of corpora, lexicon, modifiers, and tools are a few factors that make this task so difficult. These issues are solved when there is a need to identify the proper publication venues and means. Therefore, this section presents a knowledge base of publication sources, geographical distribution, types, publication channel, and year-wise distribution for the Urdu-based SA studies. Figure 4 outlines the numbers of publications according to year-wise manner. Meanwhile, only eight studies have been considered since 2012 to include the research trends in these years as well. From the total of sixty, the rest of the studies were included from 2017 and onwards.

On the other hand, Table 6 presents that the highest number of publications are journal published studies, and conferences publications are the second-highest number of studies included for performing the systematic literature review. Additionally, Table 7 presents the overall geographical distribution of the selected studies included in this survey. The addressed area of science is related to the Urdu language that is widely spoken and understood in Asia. Therefore, the maximum number of studies mapped to this region. However, several studies differ from this and are, therefore, included in Table 7.

Additionally, the quality score assigned to each study can be observed from Appendix A, which outlines the classification of the selected studies for the systematic literature review. We have classified these studies based on research type, empirical research, and methodology. Furthermore, the quality assessment score discussed earlier in this study are used to score the studies, therefore, presenting the Appendix A and Appendix B with all the required information. The research type and empirical classification are further classified, including primary research, surveys, SLRs, experimental research, and evaluation studies. These classifications have assisted us greatly in developing the research taxonomy that is discussed in the coming sections. Furthermore, we have identified the most popular publication channels in Table 7 and Table 8 that present the number of publications in the respective journals and conferences. Additionally, these studies are also classified with respect to script used in studies. The codes including “U” and “RU” are assigned for “Urdu Script” and “Roman Urdu Script” respectively. Future researchers can utilize this knowledge to explore more relevant literature.

### Assessment of RQ2: What are the primary studies that have studied and discussed the use of machine learning methods for sentiment analysis?

Machine learning methods and techniques are often used to perform complex mathematical functions (Khattak et al., 2021). These algorithms have been employed to complete different sentiment analysis tasks such as word segmentation, text cleaning, spell checking, speech tagging, and corpus and lexicon development in recent years. Additionally, the literature is enriched with studies that have addressed the issue of sentiment classification by using machine learning methods. The Appendix section outlines the studies that employed ML and DL-based models for the task of SA.

**Table 6:** Percentage of publication type.

	Publications (in percentage)
Journals	62
Conferences	36
Workshops	2

### Assessment of RQ3: What are the major challenges and opportunities for the Urdu-based sentiment analysis?

In addition to the studies outlining the different ML and DL-assisted models for the task of SA, at the same time, various studies list the opportunities and challenges that the researchers face in the field of Urdu SA. This section of the study outlines the studies that discuss the different shortcomings and opportunities for the area of Urdu-based SA. The existing literature regarding Urdu-based sentiment analysis, when summarized, reflects the various challenges such as non-availability of open access Urdu corpora (Raza et al., 2019), limitation of existing language constructs for the Urdu language like (slangs and emotions-based sentences) (Marrese-Taylor, Velásquez & Bravo-Marquez, 2014), a limited collection of negation and revised modifiers (Mehmood et al., 2019a). The need for more domain-centric words for Urdu language (Khattak et al., 2021; Hasan et al., 2018).

**Table 7:** Distribution of selected studies.

	Distribution of articles (in percentage)
America	12
Europe	18
Asia	60
Other	10

**Table 8:** Quality assessment score.

References	Score	Total
Naqvi, Majid & Abbas (2021), Basiri et al. (2021), Minaee et al. (2021) and Dong & Lian (2021)	8	5
Safder et al. (2021), Mukhtar & Khan (2020), Naqvi et al. (2020), Asif et al. (2020), Badaro et al. (2019) and Bibi et al. (2019)	7	8
Basiri, Naghsh-Nilchi & Ghassem-Aghaee (2014), Mehmood, Essam & Shafi (2018), Majeed, Mujtaba & Beg (2020) and Khattak et al. (2021)
Mehmood et al. (2020), Basiri et al. (2020), Pourpanah et al. (2020), Raza et al. (219)Lin & He (2009), Zhou et al. (2017), Sattar & Fatima (2021), Hassan et al. (2019) and Jena (2019)	6	14
Seo et al. (2020), Ali et al. (2021), Mukhtar, Khan & Chiragh (2018), Ghulam et al. (2019), Mehta & Pandya (2020), Hemmatian & Sohrabi (2019), Marrese-Taylor, Velásquez & Bravo-Marquez (2014), Babu, Kumari & Kamakshaiah (2017), Idrees et al. (2019), Altrabsheh, Cocea & Fallahkhair (2014) and Mehmood et al. (2019a)	5	13

### Assessment of RQ4. What are the most effective machine learning-based methods for performing Urdu-based SA?

The RQ4 evaluates the existing studies that have proposed and employed different ML and DL-based methods for Urdu-based SA. Appendix B outlines the validation metrics that are employed in the reviewed studies. Our findings suggest that the effectiveness of different SA methods and techniques is evaluated by considering various parameters such as achieved accuracy, recall, F-1 score, specificity, etc.

### Discussion and future directions and questions for primary studies

We have reviewed many studies, multiple Urdu-based sentiment analysis approaches, and various machine learning methods for carrying out this systematic literature review. In summary, 40 studies were shortlisted considering the defined inclusion/exclusion criteria and quality assessment scoring. In addition, we have applied a unique coding scheme for these selected studies where the studies are coded for different categories. In this way, we have assigned the code M for all the studies focusing only on at least one or more machine learning methods for sentiment analysis. Studies are coded using D which addresses one or more deep learning methods. Similarly, other similar codes such as UM, UD, “UL”, C, CO are adopted for better classification and incorporation of studies in the SLR. These are associated with Urdu-based machine learning approaches, Urdu-based Deep Learning approaches, Urdu-based Lexicon based approaches, Challenges, and Challenges and Opportunities respectively. Finally, the scrutiny of these studies is carried out by assessing and analyzing their outputs, aims, methodologies, areas of discussion, and limitations.

**Figure 5:**
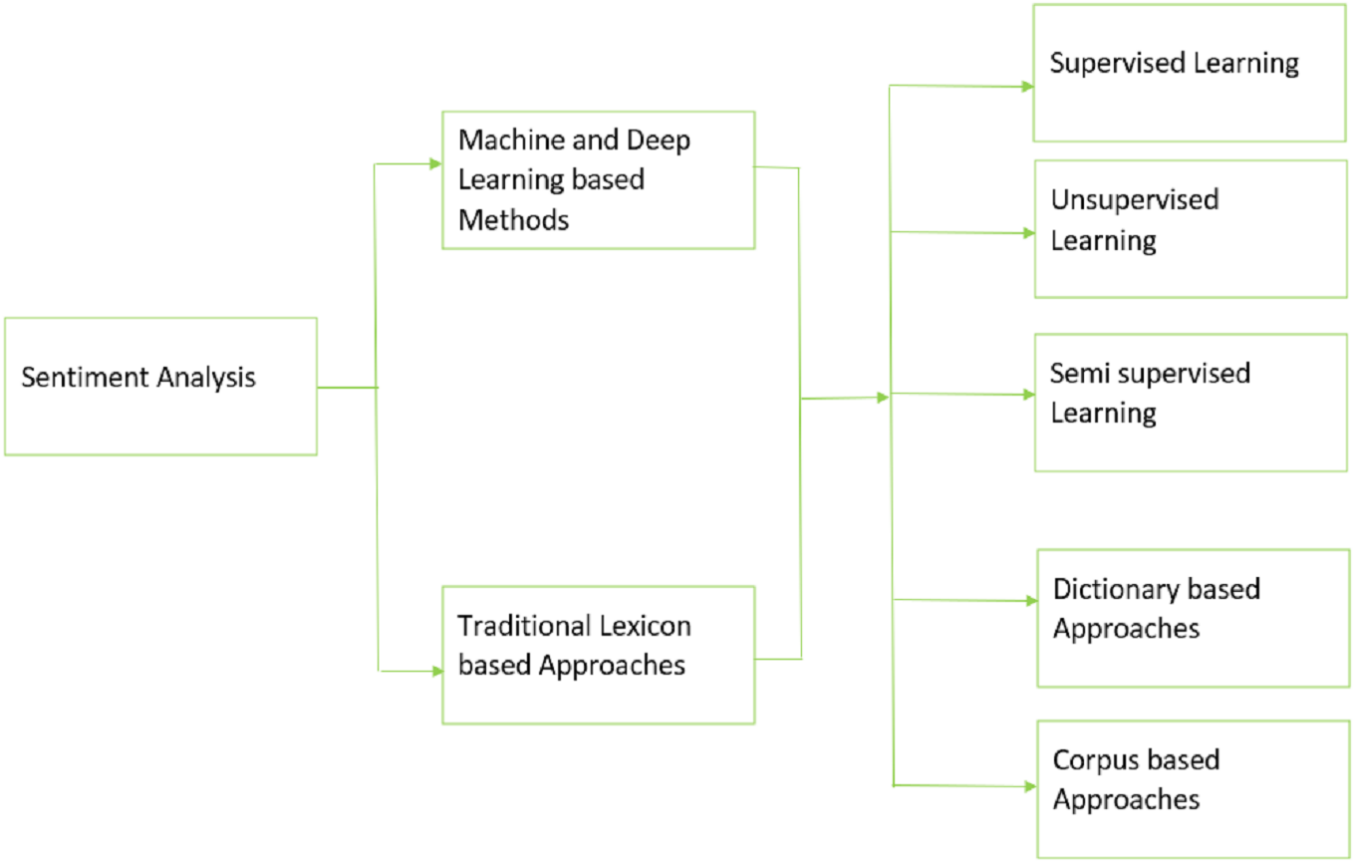
Taxonomy for the SLR.

Meanwhile, various machine and deep learning methods have been discussed to address the defined research questions in section IV. Additionally, the literature indicates that the need for pre-trained models on huge Urdu corpus, sparser, limited datasets, and lack of Urdu-based constructs remain significant issues in the field of Urdu-based sentiment analysis. The taxonomy in the Fig. 5 outlines the different techniques that are used to perform sentiment analysis in the reviewed studies. Our research indicates that lexicon-based approaches mainly include dictionary-based methods and corpus-based methods whereas machine and deep learning-based approaches include supervised, unsupervised, or semi supervised learning methods. Future work includes addressing the area of sentiment analysis of Urdu with the classification of Arabic and English scripts covering associated challenges and opportunities.

#### Questions for primary study

In the light of a systematic literature review, we report the following shortcomings in existing research.

• How can include the classification of Urdu idioms and proverbs be enhanced by employing pre-processing techniques to facilitate the process of Urdu-based sentiment analysis?
• As word sense disambiguation is the critical issue causing hurdles in Urdu SA, future researchers can focus on finding the supervised, unsupervised, and reinforcement learning-based classification techniques for classification of word meaning in the sentence.
• The literature lacks in presenting a robust public corpus of significant volume that can be utilized to train the existing deep learning models. Future researchers can develop such a corpus with several classes used in emotion detection and classification.
• Meanwhile, the concept-level sentiment analysis that keeps in mind the history of the communication needs to be explored in the future. Future researchers are suggested to explore different SA techniques to assist the context-level sentiment analysis.
• Why machine and deep learning methods have performed well on Roman Urdu datasets and how performance changes when applied to Urdu corpora.

## Conclusion

In this systematic literature review, we have explored sentiment analysis while focusing on the machine and deep learning-based methods and models that are trained and validated using Urdu corpus. While a significant number of studies and researchers have explored this domain, at the same time, the findings of this study suggest that various shortcomings exist to this day for Urdu-based sentiment analysis. This study indicated that the most relevant publication channels, included high IF journals such as ACM transactions, International Journal for Asian Languages, and IEEE journals. Furthermore, our investigation of the existing literature reflects that sentiment classification performance can be improved by overcoming the challenges such as word sense disambiguation, robust and large datasets, Urdu-based language constructs including language parsers and emoticons, context-level sentiment analysis techniques, pre-processing methods, and lexical resources. Future work includes addressing the area of sentiment analysis of Urdu with the classification of Arabic and English scripts covering associated challenges and opportunities.

##  Supplemental Information

10.7717/peerj-cs.1032/supp-1 Supplemental Information 1 Summaries of the studies

## Appendix A

See Table A1.

**Table A1:** Classification of the selected studies.

**Ref.**	Classification					Quality assessment					
	**P_Channel**	**Year**	**Research** **type**	**Empirical** **type**	**Methodology/Task**	**A**	**B**	**C**	**D**	**E**	**Total** **score**
Asghar et al. (2019)	Journal	2019	Primary	Experimental	Urdu SA	1	0	1	0	4	6
Hasan et al. (2018)	Journal	2018	Primary	Experimental	ML based SA	0	1	1	1	3	6
Khan et al. (2021b)	Journal	2021	Primary	Experimental	Deep Learning Based SA	0	1	1	0	4	6
Ghulam et al. (2019)	Journal	2019	Primary	Experimental	DL based SA	0	1	1	0	3	5
Khattak et al. (2021)	Journal	2020	Survey	No	SLR	1	0	0	1	4	6
Mehmood et al. (2019b)	Journal	2019	Primary	Experimental	ML based SA	0	1	1	0	4	6
Basiri, Naghsh-Nilchi & Ghassem-Aghaee (2014)	Conference	2014	Primary	Experimental	Supervised ML based SA in Persian	0	1	1	0	3	5
Mehmood, Essam & Shafi (2018)	Journal	2019	Primary	Experimental	ML based SA	0	1	1	1	4	7
Majeed, Mujtaba & Beg (2020)	Conference	2020	Primary	Solution article	ML based SA	0	1	0	1	3	5
Tabassum et al. (2021)	Journal	2021	Primary	Experimental	ML based SA	1	1	1	1	3	7
Mehmood et al. (2020)	Journal	2021	Primary	Experimental	ML based SA	1	1	1	0	4	7
Seo et al. (2020)	Journal	2020	Comparative study	Survey article	Deep learning models	1	1	1	1	4	8
Safder et al. (2021)	Journal	2021	Primary	Experimental	ML based SA	1	1	1	1	4	8
Ali et al. (2021)	Journal	2021	Primary	Experimental	ML based Hate Speech Analysis	1	1	1	0	4	7
Mukhtar & Khan (2020)	Journal	2020	Primary	Experimental	Lexicon based Sentiment Analysis	1	0	1	0	4	6
Naqvi et al. (2020)	Journal	2020	Primary	Experimental	ML based SA	0	1	1	1	3	6
Naqvi, Majid & Abbas (2021)	Journal	2021	Primary	Experimental	Deep Learning based SA	1	1	1	1	4	8
Basiri et al. (2021)	Journal	2021	Primary	Exploratory	CNN-RNN based SA	1	1	1	1	4	8
Asif et al. (2020)	Journal	2020	Primary	Experimental	Hate Speech using ML	0	1	1	1	4	7
Basiri et al. (2020)	Journal	2020	Primary	Experimental	ML and deep learning based review classification	0	1	1	0	3	5
Pourpanah et al. (2020)	Journal arxiv	2020	Survey	Survey article	Survey of DL Methods	0	1	0	1	3	5
Minaee et al. (2021)	Journal ACM	2021	Survey	Survey article	DL based text classification	0	1	0	0	4	5
Dong & Lian (2021)	Journal Elsevier	2021	Review	Review article	Opinion Analysis	0	1	0	1	3	5
Badaro et al. (2019)	Journal ACM	2019	Survey	Survey article	Opinion Mining	0	1	0	1	4	6
Mukhtar, Khan & Chiragh (2018)	Journal Telematics and informatics	2018	Comparative study	Experimental	Lexicon and ML approaches	1	1	1	0	3	6
Ghulam et al. (2019)	Journal Procedia CS	2019	Primary	Experimental	DL based Sentiment Analysis	0	1	1	0	3	5
Mehta & Pandya (2020)	Journal	2020	Survey	Survey article	Sentiment Analysis methods	0	1	0	1	3	5
Hemmatian & Sohrabi (2019)	Journal AI Review	2019	Survey	Survey article	Opinion mining and approaches	0	1	0	1	4	6
Raza et al. (2019)	Journal IJACSA	2019	Primary	Experimental	Text analysis for sentiment	0	1	1	1	2	5
Lin & He (2009)	Conference ACM	2009	Survey	Survey article	Sentiment Analysis	0	1	0	1	3	5
Marrese-Taylor, Velásquez & Bravo-Marquez (2014)	Journal Expert Sys	2014	Primary	Experimental	Aspect based opinion mining	0	1	0	1	4	6
Babu, Kumari & Kamakshaiah (2017)	Conference	2017	Primary	Experimental	ML based SA	0	1	1	1	2	5
Zhou et al. (2017)	Conference	2017	Primary	Experimental	Context based topic modeling	0	1	1	1	3	6
Idrees et al. (2019)	Conference	2019	Primary	Experimental	Urdu-based SA	1	1	1	1	3	7
Altrabsheh, Cocea & Fallahkhair (2014)	Conference	2019	Primary	Experimental	Feedback analysis	0	1	1	1	3	6
Sattar & Fatima (2021)	Journal Pakistan JS	2021	Primary	Experimental	Review SA	0	1	1	1	3	6
Mehmood et al. (2019a)	Journal IEEE Access	2019	Primary	Experimental	Roman Urdu SA	0	1	1	1	4	7
Hassan et al. (2019)	Journal IJST	2019	Primary	Experimental	Text classification-based SA	0	1	1	1	2	5
Nazir et al. (2020)	Conference IEEE	2020	Primary	Experimental	Roman Urdu SA	0	1	1	0	2	4
Bibi et al. (2019)	Conference IEEE	2019	Primary	Experimental	ML based Urdu SA	1	1	1	1	3	7
Jena (2019)	Journal	2019	Primary	Experimental	Emotion Analysis	0	1	1	0	3	5

## Appendix B

See Table B1.

**Table B1:** Validation classification of studies.

**Ref.**	**Classification**		
	**Validation metric**	**Theme**	**Script**
Asghar et al. (2019)	Precision, Accuracy, Recall, F-measure	UL	U
Hasan et al. (2018)	Accuracy	UM, CO	RU
Khan et al. (2021b)	Precision, Accuracy, Recall, F-measure	UD	RU
Ghulam et al. (2019)	Precision, Accuracy, Recall, F-measure	UD	RU
Khattak et al. (2021)	Survey	CO	U
Mehmood et al. (2019b)	Accuracy Unigram	UM	RU
Basiri, Naghsh-Nilchi & Ghassem-Aghaee (2014)	F-score of features	UM, CO	RU
Mehmood, Essam & Shafi (2018)	Accuracy, F-score	UM, CO	RU
Majeed, Mujtaba & Beg (2020)	Precision, Accuracy, Recall, F-measure	UM, CO	RU
Tabassum et al. (2021)	Precision, Accuracy, Recall, F-measure	UM, CO	U
Mehmood et al. (2020)	Precision, Accuracy, Recall, F-measure	UM	U
Seo et al. (2020)	Accuracy for features	UD, CO	U
Safder et al. (2021)	Precision, Accuracy, Recall, F-measure	UM, CO	U
Ali et al. (2021)	Micro F1 score	UM	U
Mukhtar & Khan (2020)	Accuracy	UL	U
Naqvi et al. (2020)	Accuracy	UM, CO	RU
Naqvi, Majid & Abbas (2021)	Precision, Accuracy, Recall, F-measure	UD, CO	U
Basiri et al. (2021)	Precision, Accuracy, Recall, F-measure	UD, CO	U
Asif et al. (2020)	Precision, Recall, F-measure	UM, CO	RU
Basiri et al. (2020)	Accuracy	UM, UD	RU
Pourpanah et al. (2020)	F-measure	UD, CO	RU
Minaee et al. (2021)	Survey	UD	RU
Dong & Lian (2021)	Review	UL, CO	RU
Badaro et al. (2019)	survey	UL, CO	RU
Mukhtar, Khan & Chiragh (2018)	Precision, Recall, F-measure	UL	U
Ghulam et al. (2019)	Precision, Accuracy, Recall, F-measure	UD	RU
Mehta & Pandya (2020)	Review	UL, CO	RU
Hemmatian & Sohrabi (2019)	Survey	UM, CO	RU
Raza et al. (2019)	Accuracy, F-measure	UL, CO	RU
Lin & He (2009)	Accuracy	UM, CO	RU
Marrese-Taylor, Velásquez & Bravo-Marquez (2014)	Accuracy, F-measure	UM, CO	RU
Babu, Kumari & Kamakshaiah (2017)	F-measure	UD, CO	RU
Zhou et al. (2017)	Accuracy	ML, CO	U
Idrees et al. (2019)	Accuracy	UD, CO	RU
Altrabsheh, Cocea & Fallahkhair (2014)	Precision, Accuracy, Recall, F-measure	UL, CO	RU
Sattar & Fatima (2021)	Accuracy, F-measure	UM, CO	RU
Mehmood et al. (2019a)	F-score of features	UM, CO	RU
Hassan et al. (2019)	Accuracy	UL, CO	RU
Nazir et al. (2020)	Precision, Accuracy, Recall, F-measure	UM, CO	U
Bibi et al. (2019)	Accuracy	UD, CO	RU
Jena (2019)	Precision, Accuracy, Recall, F-measure	UL,CO	U

## Additional Information and Declarations

### Data Availability

The following information was supplied regarding data availability: The detailed summaries of the reviewed studies is available in the Supplemental File.

## References

[ref-1] Ali MZ, Rauf S, Javed K, Hussain S (2021). Improving hate speech detection of urdu tweets using sentiment analysis. IEEE Access.

[ref-2] Altrabsheh N, Cocea M, Fallahkhair S (2014). Learning sentiment from students feedback for real-time interventions in classrooms.

[ref-3] Anwar W, Wang X, Wang X-l (2006). A survey of automatic Urdu language processing.

[ref-4] Asghar MZ, Khan A, Bibi A, Kundi FM, Ahmad H (2017). Sentence-level emotion detection framework using rule-based classification. Cognitive Computation.

[ref-5] Asghar MZ, Sattar A, Khan A, Ali A, Masud Kundi F, Ahmad S (2019). Creating sentiment lexicon for sentiment analysis in Urdu: the case of a resource-poor language. Expert Systems.

[ref-6] Asif M, Ishtiaq A, Ahmad H, Aljuaid H, Shah J (2020). Sentiment analysis of extremism in social media from textual information. Telematics and Informatics.

[ref-7] Awais DM, Shoaib DM (2019). Role of discourse information in Urdu sentiment classification: a rule-based method and machine-learning technique. ACM Transactions on Asian and Low-Resource Language Information Processing (TALLIP).

[ref-8] Babu AG, Kumari SS, Kamakshaiah K (2017). An experimental analysis of clustering sentiments for opinion mining.

[ref-9] Badaro G, Baly R, Hajj H, El-Hajj W, Shaban KB, Habash N, Al-Sallab A, Hamdi A (2019). A survey of opinion mining in Arabic: a comprehensive system perspective covering challenges and advances in tools, resources, models, applications, and visualizations. ACM Transactions on Asian and Low-Resource Language Information Processing (TALLIP).

[ref-10] Basiri ME, Abdar M, Cifci MA, Nemati S, Acharya UR (2020). A novel method for sentiment classification of drug reviews using fusion of deep and machine learning techniques. Knowledge-Based Systems.

[ref-11] Basiri ME, Naghsh-Nilchi AR, Ghassem-Aghaee N (2014). A framework for sentiment analysis in persian. Open Transactions on Information Processing.

[ref-12] Basiri ME, Nemati S, Abdar M, Cambria E, Acharya UR (2021). ABCDM: an attention-based bidirectional CNN-RNN deep model for sentiment analysis. Future Generation Computer Systems.

[ref-13] Bibi R, Qamar U, Ansar M, Shaheen A (2019). Sentiment analysis for Urdu news tweets using decision tree.

[ref-14] Brereton P, Kitchenham BA, Budgen D, Turner M, Khalil M (2007). Lessons from applying the systematic literature review process within the software engineering domain. Journal of Systems and Software.

[ref-15] Dashtipour K, Poria S, Hussain A, Cambria E, Hawalah AY, Gelbukh A, Zhou Q (2016). Multilingual sentiment analysis: state of the art and independent comparison of techniques. Cognitive Computation.

[ref-16] Dong X, Lian Y (2021). A review of social media-based public opinion analyses: challenges and recommendations. Technology in Society.

[ref-17] Fernandez A, Insfran E, Abrahão S (2011). Usability evaluation methods for the web: a systematic mapping study. Information and Software Technology.

[ref-18] Ghulam H, Zeng F, Li W, Xiao Y (2019). Deep learning-based sentiment analysis for Roman Urdu text. Procedia Computer Science.

[ref-19] Hasan A, Moin S, Karim A, Shamshirband S (2018). Machine learning-based sentiment analysis for twitter accounts. Mathematical and Computational Applications.

[ref-20] Hassan SM, Ali F, Wasi S, Javeed S, Hussain I, Ashraf SN (2019). Roman-urdu news headline classification with ir models using machine learning algorithms. Indian Journal of Science and Technology.

[ref-21] Hemmatian F, Sohrabi MK (2019). A survey on classification techniques for opinion mining and sentiment analysis. Artificial Intelligence Review.

[ref-22] Idrees F, Qadir J, Mehmood H, Hassan SU, Batool A (2019). Urdu language based information dissemination system for low-literate farmers.

[ref-23] Jena R (2019). Sentiment mining in a collaborative learning environment: capitalising on big data. Behaviour & Information Technology.

[ref-24] Khan IU, Khan A, Khan W, Suud MM, Alam MM, Subhan F, Asghar MZ (2021a). A review of Urdu sentiment analysis with multilingual perspective: a case of Urdu and roman Urdu language. Computers.

[ref-25] Khan L, Amjad A, Ashraf N, Chang H-T, Gelbukh A (2021b). Urdu sentiment analysis with deep learning methods. IEEE Access.

[ref-26] Khan W, Daud A, Nasir JA, Amjad T, Arafat S, Aljohani N, Alotaibi FS (2019). Urdu part of speech tagging using conditional random fields. Language Resources and Evaluation.

[ref-27] Khattak A, Asghar MZ, Saeed A, Hameed IA, Hassan SA, Ahmad S (2021). A survey on sentiment analysis in Urdu: a resource-poor language. Egyptian Informatics Journal.

[ref-28] Kitchenham B (2004). Procedures for performing systematic reviews. Keele, UK, Keele University.

[ref-29] Lin C, He Y (2009). Joint sentiment/topic model for sentiment analysis.

[ref-30] Liu B (2012). Sentiment analysis and opinion mining. Synthesis Lectures on Human Language Technologies.

[ref-31] Lo SL, Cambria E, Chiong R, Cornforth D (2017). Multilingual sentiment analysis: from formal to informal and scarce resource languages. Artificial Intelligence Review.

[ref-32] Majeed A, Mujtaba H, Beg MO (2020). Emotion detection in roman urdu text using machine learning.

[ref-33] Marrese-Taylor E, Velásquez JD, Bravo-Marquez F (2014). A novel deterministic approach for aspect-based opinion mining in tourism products reviews. Expert Systems with Applications.

[ref-34] Mehmood F, Ghani MU, Ibrahim MA, Shahzadi R, Mahmood W, Asim MN (2020). A precisely xtreme-multi channel hybrid approach for roman urdu sentiment analysis. IEEE Access.

[ref-35] Mehmood K, Essam D, Shafi K (2018). Sentiment analysis system for Roman Urdu.

[ref-36] Mehmood K, Essam D, Shafi K, Malik MK (2019a). Discriminative feature spamming technique for roman urdu sentiment analysis. IEEE Access.

[ref-37] Mehmood K, Essam D, Shafi K, Malik MK (2019b). Sentiment analysis for a resource poor languageRoman Urdu. ACM Transactions on Asian and Low-Resource Language Information Processing (TALLIP).

[ref-38] Mehta P, Pandya S (2020). A review on sentiment analysis methodologies, practices and applications. International Journal of Scientific and Technology Research.

[ref-39] Minaee S, Kalchbrenner N, Cambria E, Nikzad N, Chenaghlu M, Gao J (2021). Deep learning—based text classification: a comprehensive review. ACM Computing Surveys (CSUR).

[ref-40] Mukhtar N, Khan MA (2020). Effective lexicon-based approach for Urdu sentiment analysis. Artificial Intelligence Review.

[ref-41] Mukhtar N, Khan MA, Chiragh N (2018). Lexicon-based approach outperforms supervised machine learning approach for Urdu sentiment analysis in multiple domains. Telematics and Informatics.

[ref-42] Naqvi RA, Khan MA, Malik N, Saqib S, Alyas T, Hussain D (2020). Roman Urdu news headline classification empowered with machine learning. Computers, Materials & Continua.

[ref-43] Naqvi U, Majid A, Abbas SA (2021). UTSA: Urdu text sentiment analysis using deep learning methods. IEEE Access.

[ref-44] Nazir MK, Ahmad M, Ahmad H, Qayum MA, Shahid M, Habib MA (2020). Sentiment analysis of user reviews about hotel in Roman Urdu.

[ref-45] Ouhbi S, Idri A, Fernández-Alemán JL, Toval A (2015). Requirements engineering education: a systematic mapping study. Requirements Engineering.

[ref-46] Portal CC (2018). CORE conference portal. http://portal.core.edu.au/conf-ranks/.

[ref-47] Pourpanah F, Abdar M, Luo Y, Zhou X, Wang R, Lim CP, Wang X-Z (2020). A review of generalized zero-shot learning methods.

[ref-48] Rank SJC (2018). Scimago journal & country rank. https://www.scimagojr.com.

[ref-49] Raza H, Faizan M, Hamza A, Mushtaq A, Akhtar N (2019). Scientific text sentiment analysis using machine learning techniques. International Journal of Advanced Computer Science and Applications.

[ref-50] Safder I, Mahmood Z, Sarwar R, Hassan S-U, Zaman F, Nawab RMA, Bukhari F, Abbasi RA, Alelyani S, Aljohani NR (2021). Sentiment analysis for Urdu online reviews using deep learning models. Expert Systems.

[ref-51] Sattar A, Fatima J (2021). Sentiment analysis based on reviews using machine learning techniques. Pakistan Journal of Engineering and Technology.

[ref-52] Seo S, Kim C, Kim H, Mo K, Kang P (2020). Comparative study of deep learning-based sentiment classification. IEEE Access.

[ref-53] Syed AZ, Aslam M, Martinez-Enriquez AM (2010). Lexicon based sentiment analysis of Urdu text using SentiUnits.

[ref-54] Tabassum N, Alyas T, Hamid M, Saleem M, Malik S, Ali Z, Farooq U (2021). Semantic analysis of Urdu english tweets empowered by machine learning. Intelligent Automation and Soft Computing.

[ref-55] Wohlin C (2014). Guidelines for snowballing in systematic literature studies and a replication in software engineering.

[ref-56] Zhou X, Tao X, Rahman MM, Zhang J (2017). Coupling topic modelling in opinion mining for social media analysis.

