# Supplementary material for: Sentiment analysis techniques, challenges, and opportunities: Urdu language-based analytical study
Source: PeerJ Comput Sci. 2022 Aug 31;8:e1032. doi: 10.7717/peerj-cs.1032 (PMC9454799; doi:10.7717/peerj-cs.1032)
Supplement: Supplemental Information 1 [file peerj-cs-08-1032-s001.docx]

**Summary of the Studies**

**Paper 1**

Creating sentiment lexicon for sentiment analysis in Urdu: The case of a resource‐poor language

| Citation Information | 15 February 2019, Wiley Expert System |
| --- | --- |
| Purpose (has the author formulated a problem?) | Creation of sentiment Lexicon |
| Subjects | Urdu, Sentiment Analysis, Lexicon development |
| Methodology | word‐level translation scheme for creating a first comprehensive Urdu polarity resource |
| Design and analysis | study assigns two polarity scores, positive and negative, to each Urdu opinion word. |
| Conclusions and results | evaluation results show that the polarity scores assigned by this technique are more accurate than the baseline methods. |
| Implications | Improvement in results due to proposed methodology. |
| Weaknesses | Could not find. |
| Strengths | The improved results achieved in terms of precision, recall, and F‐measure show the efficacy of the proposed approach |
| Other (e.g. has relevant literature been evaluated? Contribution?) | Yes. |

Included.

**Paper 2**

Machine Learning-Based Sentiment Analysis for Twitter Accounts

| Citation Information | 2018, Mathematical and Computational Applications |
| --- | --- |
| Purpose (has the author formulated a problem?) | Machine Learning-Based Sentiment Analysis for Twitter Accounts |
| Subjects | Sentiment Analysis, Opinion Mining for election, Twitter Data |
| Methodology | hybrid approach that involves a sentiment analyzer that includes machine learning |
| Design and analysis | Naïve Bayes and support vector machines (SVM) |
| Conclusions and results | TextBlob and W-WSD have almost the same accuracy of sentiments, which was about 62%. |
| Implications | Improved results |
| Weaknesses | Not related to Urdu language-based opinion mining |
| Strengths | Could not find any |
| Other (e.g. has relevant literature been evaluated? Contribution?) | Yes. |

Included. Same strategy can be explored for Urdu language as well.

**Paper 3**

Urdu Sentiment Analysis with Deep Learning Methods

| Citation Information | July 2021, IEEE Access, Volume 9, 2021 |
| --- | --- |
| Purpose (has the author formulated a problem?) | Creation of a benchmark corpus, evaluation of various deep learning-based models |
| Subjects | Urdu Sentiment Analysis, Corpus Development, Deep Learning Models |
| Methodology | Evaluation of deep learning models including 1D-CNN and LSTM for Urdu Sentiment Analysis. |
| Design and analysis | Count based technique and fastText based word embeddings. |
| Conclusions and results | Count based n-gram technique produced higher accuracy of 82.05%. |
| Implications | The deep learning models tend to provide the higher accuracy as compared to other techniques. |
| Weaknesses | Only positive and negative classification. |
| Strengths | the study provided solid arguments against all the proposed claims. |
| Other (e.g. has relevant literature been evaluated? Contribution?) | Yes. |

Included.

**Paper 4**

Deep Learning-Based Sentiment Analysis for Roman Urdu Text

| Citation Information | 2019, Procedia Computer Science 147 (2019) |
| --- | --- |
| Purpose (has the author formulated a problem?) | Deep learning based Roman Urdu Sentiment Analysis |
| Subjects | Deep learning, Sentiment Analysis, Roman Urdu, |
| Methodology | Deep LSTM |
| Design and analysis | Impact of SML algorithm in sentiment analysis of roman urdu text |
| Conclusions and results | Model performed best and surpasses the F1 and accuracy of all the Machine Learning baseline methods and achieved 0.95 Accuracy and 0.94 F1 score. |
| Implications | Improved the performance by increasing the overall model accuracy |
| Weaknesses | It is related to Roman Urdu Sentiment Analysis and Classification |
| Strengths | Improved results as compared to given baseline results. |
| Other (e.g. has relevant literature been evaluated? Contribution?) | No Literature Review Section in the Paper. |

Included. The paper would be included in the literature survey of the final draft.

**Paper 5**

A survey on sentiment analysis in Urdu: A resource-poor language

| Citation Information | 15 May 2020, Egyptian Informatics Journal |
| --- | --- |
| Purpose (has the author formulated a problem?) | Survey of Sentiment Analysis in Urdu language |
| Subjects | Sentiment Analysis, Urdu Language, Survey |
| Methodology | present state-of-art survey for identifying the progress and shortcomings saddling Urdu sentiment analysis and propose rectifications. |
| Design and analysis | categorizing the studies along three dimensions, namely: text pre-processing lexical resources and sentiment classification. |
| Conclusions and results | Progress of research in each of the three categories is reported along with outlining the shortcoming in the recent research. |
| Implications | Outlined the trends, and challenges in the Urdu sentiment analysis |
| Weaknesses | Could not find any. |
| Strengths | An evaluation of sophisticated lexical resources is carried out by the researchers.  Defined and evaluated the study using the research questions and SLR based approach. |
| Other (e.g. has relevant literature been evaluated? Contribution?) | Yes. |

Included. While the paper is a secondary study however it provides us great insights into the topic with outlining the trends in the field along with the opportunities and challenges.

**Paper 6**

Sentiment Analysis for a Resource Poor Language—Roman Urdu

| Citation Information | August 2019, ACM Trans, Vol. 19, No. 1, Article 10. |
| --- | --- |
| Purpose (has the author formulated a problem?) | Focusing on limited data set problems |
| Subjects | Roman Urdu Classification |
| Methodology | Collection of 11,000 reviews from multiple fields, multi-annotator |
| Design and analysis | World-level features, character-level features, and feature union |
| Conclusions and results | Reduced error rate by 12% as compared to baseline. |
| Implications |  |
| Weaknesses | Positive and negative classification only |
| Strengths | Reduced error rate that was encountered in previous research |
| Other (e.g. has relevant literature been evaluated? Contribution?) | Yes |

Included.

**Paper 7**

A Framework for Sentiment Analysis in Persian

| Citation Information | Volume 1, Number 3, November 2014. Open Transactions on Information Processing |
| --- | --- |
| Purpose (has the author formulated a problem?) | Sentiment Analysis in Persian language |
| Subjects | Persian Language, Sentiment Analysis, Framework |
| Methodology | Development of a Persian polarity lexicon, review of challenges, |
| Design and analysis | the performance of the proposed method in classifying the polarity of online cell phone reviews. |
| Conclusions and results | The proposed model outperformed the existing state of the art supervised learning-based machine learning models for Persian language sentiment analysis. |
| Implications | Improved results. |
| Weaknesses | The paper is not related to Urdu Sentiment Analysis. |
| Strengths | N/A |
| Other (e.g. has relevant literature been evaluated? Contribution?) | Yes. |

Excluded. The dynamics of Urdu and Persian language differ and therefore, the paper can not be used as the base study for performing Urdu based opinion mining.

**Paper 8**

Sentiment analysis system for Roman Urdu

| Citation Information | November 2018, Intelligent Computing, Springer |
| --- | --- |
| Purpose (has the author formulated a problem?) | Sentiment Analysis of Roman Urdu using review-based data |
| Subjects | Roman Urdu, Sentiment analysis, Corpus Development, Reviews |
| Methodology | Machine learning based algorithms were used to perform 36 different experiments |
| Design and analysis | unigram, bigram, and uni-bigram (unigram + bigram) features. |
| Conclusions and results | Naïve Bayes (NB) and Logistic Regression (LR) performed better than rest of the models. |
| Implications | Feature reduction improved the results. |
| Weaknesses | Conference paper with very little details and explanations. |
| Strengths | Developed a novel corpus and perform several experiments. |
| Other (e.g. has relevant literature been evaluated? Contribution?) | Yes. |

Excluded.

**Paper 9**

Emotion Detection in Roman Urdu Text using Machine Learning

| Citation Information | September 2020, ACM International Conference on Automated Software Engineering Workshops (ASEW) |
| --- | --- |
| Purpose (has the author formulated a problem?) | The use of machine learning algorithms for emotion detection in roman Urdu text |
| Subjects | Machine Learning, Emotion Detection from text, Sentiment Analysis |
| Methodology | Machine learning based text-based emotion detection using Roman Urdu text |
| Design and analysis | develop a comprehensive corpus of 18k  sentences that are gathered from different domains and annotate it  with six different classes. |
| Conclusions and results | KNN, Decision tree, SVM, and Random Forest on our corpus. results showed that the SVM model achieves a better F-measure score. |
| Implications | Improved results than baseline models. |
| Weaknesses | Could not find |
| Strengths | Improved emotion classification model. |
| Other (e.g. has relevant literature been evaluated? Contribution?) | Yes. |

Included.

**Paper 10**

Semantic Analysis of Urdu English Tweets Empowered by Machine Learning

| Citation Information | 2021, Intelligent Automation & Soft Computing |
| --- | --- |
| Purpose (has the author formulated a problem?) | Semantic Analysis of Urdu and English twitter |
| Subjects | Negative and Positive Classification of Urdu and English tweets |
| Methodology | Combining RNN with LSTM |
| Design and analysis | Machine Learning based approach |
| Conclusions and results | 87% accuracy on the Urdu dataset and 92% on the English dataset. |
| Implications | Limited Urdu Corpus. |
| Weaknesses | Positive and negative classification only. |
| Strengths | Improved results by combining RNN and LSTM |
| Other (e.g. has relevant literature been evaluated? Contribution?) | Yes |

Included.

**Paper 11**

A Precisely Xtreme-Multi Channel Hybrid Approach for Roman Urdu Sentiment Analysis

Included.

| Citation Information | Oct 2020, IEEE Access |
| --- | --- |
| Purpose (has the author formulated a problem?) | Roman Urdu Sentiment Analysis and development of benchmark dataset |
| Subjects | Roman Urdu Classification namely positive, and negative classification |
| Methodology | The study employed use of machine learning, deep learning, and hybrid deep learning approaches for classification |
| Design and analysis | Machine and deep learning approaches along with multi-channel hybrid approach |
| Conclusions and results | Achieved better accuracy by 9% for Roman Urdu classification. |
| Implications | Improved error rate for the classification. |
| Weaknesses | Binary classification only |
| Strengths | Multi-channel hybrid methodology consisting of CNN, RNN, and Neural Word Embeddings |
| Other (e.g. has relevant literature been evaluated? Contribution?) | Yes. |

**Paper 12**

Comparative Study of Deep Learning-Based Sentiment Classification

Included. Though secondary study but outlined great points in the research.

| Citation Information | Jan 2020, IEEE Access |
| --- | --- |
| Purpose (has the author formulated a problem?) | Comparative Study of Deep Learning-Based Sentiment Classification |
| Subjects | comparative study of eight deep neural networks for 13 different datasets |
| Methodology | Identifying and evaluating deep learning-based sentiment analysis systems using RNNs and CNNs for word and character level input methods |
| Design and analysis | Comparative analysis of existing deep learning-based approaches. |
| Conclusions and results | The research indicates that deep learning-based approaches provide better results as compared to machine learning based approaches. |
| Implications | The study emphasis on incorporating deep learning-based models to ensure higher classification accuracies |
| Weaknesses | The study dictates that the existing approaches lack developing and training models from scratch to assess the performance. |
| Strengths | The study evaluated the existing benchmarked systems for two different inputs namely word level input and character level input. |
| Other (e.g. has relevant literature been evaluated? Contribution?) | Yes. |

**Paper 13**

Sentiment analysis for Urdu online reviews using deep learning models

Included.

| Citation Information | May 2021, Expert Systems |
| --- | --- |
| Purpose (has the author formulated a problem?) | The study is aimed towards sentiment analysis of Urdu online reviews |
| Subjects | Online Reviews on websites, social media, blogs, are analyzed and evaluated for sentiment analysis |
| Methodology | Machine learning based models have been adopted to perform binary and ternary classification of online Urdu Reviews. |
| Design and analysis | LSTM, RCNN, N-gram, SVMs have been used to perform opinion mining. |
| Conclusions and results | The models outperformed the existing models with accuracy of 84.98%. |
| Implications | Improved classification. |
| Weaknesses | The study lacked in terms of specifying the world or character level input and aimed to incorporate these features in coming future. |
| Strengths | The study involved humanly annotated corpus of 10,008 reviews from fields. |
| Other (e.g. has relevant literature been evaluated? Contribution?) | Yes. |

**Paper 14**

| Citation Information | June 2021, IEEE Access |
| --- | --- |
| Purpose (has the author formulated a problem?) | The study is aimed towards hate speech detection in Urdu Tweets on microblogging data of social media platform twitter |
| Subjects | Urdu Hate speech Detection |
| Methodology | Machine learning based approaches to solve sparsity, dimensionality, and class imbalance problems. |
| Design and analysis | SVM and MNV algorithms are utilized for achieving desired results. |
| Conclusions and results | Improved detection of Urdu hate speech |
| Implications | The adopted approach resulted in improved detection of hate speech. |
| Weaknesses | The authors aimed to assess the performance of deep learning algorithms also in the future as well. |
| Strengths | The study validated their claims by removing the sparsity, dimensionality, and class imbalance problems. |
| Other (e.g. has relevant literature been evaluated? Contribution?) | Yes. |

Improving Hate Speech Detection of Urdu Tweets Using Sentiment Analysis

Included.

**Paper 15**

Effective lexicon‑based approach for Urdu sentiment analysis

Included.

| Citation Information | 2019, Effective lexicon‑based approach for Urdu sentiment analysis, Springer, Artificial Intelligence Review (2020) 53:2521–2548 |
| --- | --- |
| Purpose (has the author formulated a problem?) | Urdu Sentiment Analysis using Urdu Blogs |
| Subjects | Lexicon based approach, Urdu sentiment analysis, |
| Methodology | The study included verbs, context-dependent words, and intensifiers along with the traditionally used nouns, adjectives, and negations. |
| Design and analysis | Urdu based Sentiment Analysis by incorporating improved lexicon approach |
| Conclusions and results | The study outperformed the existing studies with 89.03% accuracy with 0.86 precision, 0.90 recall and 0.88 F-measure. |
| Implications | As a result of using improved lexicon-based approach for Urdu classification higher efficiency is observed. |
| Weaknesses | The authors aimed to improve the classification in future by adding more classes apart from negative and positive. |
| Strengths | Incorporation of Urdu Sentiment Lexicon and analyzer that significantly improved the results. |
| Other (e.g. has relevant literature been evaluated? Contribution?) | Yes. |

**Paper 16**

Roman Urdu News Headline Classification Empowered with Machine Learning

Included.

| Citation Information | 2020, Computers, Materials & Continua, CMC, vol.65, no.2, pp.1221-1236, 2020 |
| --- | --- |
| Purpose (has the author formulated a problem?) | Roman Urdu News Headline Classification |
| Subjects | Machine Learning, Roman Urdu, News Classification |
| Methodology | Data Collection from various news websites and social media, and using machine learning |
| Design and analysis | Machine Learning techniques (CNN, LSTM, LR and MNB) for classification of Urdu news headline into five major categories. |
| Conclusions and results | The study successfully managed to outperform the existing studies by producing higher accuracy. |
| Implications | The improved lexicon technique along with the use of machine learning based algorithms resulted in better classification. |
| Weaknesses | The technique relied heavily on word level feature extraction and did not explore other features such as character level feature extraction. |
| Strengths | Multi nominal Naïve Bays model achieved accuracy of 90.17% for roman Urdu news classification. |
| Other (e.g. has relevant literature been evaluated? Contribution?) | Yes. |

**Paper 17**

UTSA: Urdu Text Sentiment Analysis Using Deep Learning Methods

Included.

| Citation Information | August 2021, IEEE Access, Volume 9, 2021 |
| --- | --- |
| Purpose (has the author formulated a problem?) | Effect of using deep learning approaches for Urdu Text Sentiment Analysis Classification |
| Subjects | Urdu text Sentiment Analysis, deep learning models, unsupervised learning |
| Methodology | LSTM, Bi-LSTM, DCNN |
| Design and analysis | Deep learning-based models along with pretrained models and unsupervised learning techniques |
| Conclusions and results | Bi-LSTM outperformed other models by achieving higher accuracy of 77.9%. |
| Implications | The incorporation of deep learning models comparatively increased the accuracy for the emotion classification. |
| Weaknesses | I could not find any. |
| Strengths | The study provides argument that deep learning based approaches can be employed for improving the existing sentiment analysis-based systems. |
| Other (e.g. has relevant literature been evaluated? Contribution?) | Yes. |

**Paper 18**

ABCDM: An Attention-based Bidirectional CNN-RNN Deep Model for sentiment analysis

| Citation Information | March 2020, Elsevier |
| --- | --- |
| Purpose (has the author formulated a problem?) | Attention based Sentiment Analysis |
| Subjects | Deep Learning, CNN, LSTM, Sentiment Analysis, Attention mechanism |
| Methodology | Attention-based Bidirectional CNN-RNN Deep Model (ABCDM) |
| Design and analysis | Incorporated two independent bidirectional LSTM and GRU layers |
| Conclusions and results | Outperformed the previous studies by obtaining highest accuracy |
| Implications | the comparison of the results obtained for review and tweet datasets shows that the amount of improvements on short tweet datasets is less than the similar case for the long review datasets. |
| Weaknesses | Not related to Urdu Sentiment Analysis |
| Strengths | focused on polarity detection in document-level  sentiment analysis using ABCDM |
| Other (e.g. has relevant literature been evaluated? Contribution?) | Yes |

Included. Same could be applied for resource poor language such as Urdu.

| Citation Information | January 2020, Elsevier Journals |
| --- | --- |
| Purpose (has the author formulated a problem?) | Extremism detection in social media |
| Subjects | Multilingual, SVMs, Extremism, CNN, Sentiment Analysis |
| Methodology | research focuses on the sentimental  analysis of social media multilingual textual data to discover the intensity of the sentiments of extremism. |
| Design and analysis | multilingual lexicon with the intensity weights along with the use of Naïve Bayes and Linear Support Vector Classifier algorithms |
| Conclusions and results | The model achieved accuracy of 82%. |
| Implications | Could not found |
| Weaknesses | Not related to Urdu Sentiment Analysis |
| Strengths | Roman Urdu, English, multilingual Sentiment Analysis |
| Other (e.g. has relevant literature been evaluated? Contribution?) | Yes |

**Paper 19**

Sentiment analysis of extremism in social media from textual information

Included.

| Citation Information | April 2020, Knowledge Base Systems, Elsevier Journals |
| --- | --- |
| Purpose (has the author formulated a problem?) | Classification of Drug Reviews |
| Subjects | Machine Learning, Deep Learning, Sentiment Analysis, Drug Reviews |
| Methodology | The study employed the use of 3-way fusion of three deep models with a traditional model (3W3DT) |
| Design and analysis | two deep fusion models based on three-way decision theory to analyze the drug reviews. |
| Conclusions and results | Outperformed the previous models by 4% accuracy. |
| Implications | Could not found. |
| Weaknesses | Meanwhile the paper provided new insights into the topic however, it is not related to the Urdu text Classification. |
| Other (e.g. has relevant literature been evaluated? Contribution?) | Yes |

**Paper 20**

A novel method for sentiment classification of drug reviews using fusion of deep and machine learning techniques

Included. Impact of the employed methodology can be explored for opinion mining and intent analysis in urdu as well.

**Paper 21**

TClustVID: A novel machine learning classification model to investigate topics and sentiment in COVID-19 tweets

Included. Could be replicated for Urdu Corpus.

| Citation Information | May 2021, Knowledge Based System, Elsevier Journals |
| --- | --- |
| Purpose (has the author formulated a problem?) | Topic Discovery and Sentiment Analysis of Covid-related microblogging data from twitter |
| Subjects | Topic discovery, sentiment analysis, machine learning, CNN |
| Methodology | Machine Learning based topic discovery and sentiment analysis |
| Design and analysis | intelligent clustering-based classification and topic extracting model named TClustVID that analyzes COVID-19-related public tweets to extract significant sentiments with high accuracy. |
| Conclusions and results | TClustVID showed higher performance  compared to traditional methodologies that are determined by clustering criteria |
| Implications | This model is helpful to identify important  themes about the situation at the time the tweets were sent, and can enable designing better strategies to counter the  pandemic that takes human responses and behavior into account. |
| Weaknesses | Not related to Urdu text Sentiment Analysis and Classification |
| Strengths | Robust classification and improved sentiment analysis on urdu data |
| Other (e.g. has relevant literature been evaluated? Contribution?) | Yes |

**Paper 22**

Cross-SEAN: A cross-stitch semi-supervised neural attention model for COVID-19 fake news detection

| Citation Information | April 2021, Applied Soft Computing, Elsevier Journals |
| --- | --- |
| Purpose (has the author formulated a problem?) | Fake News Detection |
| Subjects | Semi Supervised Learning, Fake News Detection, Covid-19 |
| Methodology | Cross-SEAN, a cross-stitch-based semi supervised end-to-end neural attention model which leverages the large amount of unlabeled data. |
| Design and analysis | The study introduced CTF, a large-scale COVID-19 Twitter dataset with labelled genuine and fake tweets. |
| Conclusions and results | outperformed the best baseline by 9%. |
| Implications | develop Chrome-SEAN, a Cross-SEAN based chrome extension for real-time detection of fake tweets. |
| Weaknesses | Not related to Urdu language Sentiment Analysis. |
| Other (e.g. has relevant literature been evaluated? Contribution?) | The study aimed to add additional improved filters to the process of extracting external knowledge to remove possible bias and  noise. |

Excluded.

| Citation Information | 2020, IEEE Access |
| --- | --- |
| Purpose (has the author formulated a problem?) | Emotion Detection Using Sentiment Analysis and Deep Learning |
| Subjects | Behavioral Analysis, Emotion Detection, Opinion Mining, Sentiment Analysis, DCNN |
| Methodology | Deep LSTM using Sentiment140 Dataset and emotional tweet datasets |
| Design and analysis | six primary emotions classification - joy,  surprise, sad, fear, anger, and disgust |
| Conclusions and results | Outperformed the baseline models on Sentiment140 corpus |
| Implications | ELMo is a large scale contextsensitive  word embedding model that can be explored in the future to improve the performance of classifiers A, B and C in the proposed model |
| Weaknesses | Not related to Urdu text Sentiment Analysis |
| Strengths | Improved Classification for English Language based Emotion Detection |
| Other (e.g. has relevant literature been evaluated? Contribution?) | YES |

**Paper 23**

Cross-Cultural Polarity and Emotion Detection Using Sentiment Analysis and Deep Learning on COVID-19 Related Tweets

Included. Same could be applied on Urdu Corpus.

| Citation Information | Feb 2020, Springer Journals |
| --- | --- |
| Purpose (has the author formulated a problem?) | Sentiment Analysis of comments from YouTube driven corpus comprising of Roman Hindi and English text |
| Subjects | Emotion Detection, Opinion Mining, Hope Speech Detection, Sentiment Analysis |
| Methodology | Polyglot word-embeddings with deep CNN |
| Conclusions and results | Improved classification accuracy as compared to previous studies. |
| Weaknesses | Not related to Urdu language-based opinion mining |
| Strengths | Could not found |
| Other (e.g. has relevant literature been evaluated? Contribution?) | No |

**Paper 24**

Hope Speech Detection: A Computational Analysis of The Voice of Peace

Excluded.

**Paper 25**

A Review of Generalized Zero-Shot Learning Methods

| Citation Information | 19 May 2021, arXiv: 2011.08641v3 |
| --- | --- |
| Purpose (has the author formulated a problem?) | Review of Learning methods for Sentiment Analysis |
| Subjects | Semantic Embedding, Deep Learning, GANs, Generalized zero shot learning |
| Methodology | The study presents a comprehensive review on GZSL. Firstly, we provide an overview of GZSL including the problems and challenges. |
| Design and analysis | Then, it introduces a hierarchical categorization for the GZSL methods and  discuss the representative methods in each category. |
| Conclusions and results | a hierarchical categorization of GZSL methods along with their representative models has been provided |
| Strengths | First ever comprehensive review for generalized zero shot learning methods |
| Other (e.g., has relevant literature been evaluated? Contribution?) | YES |

Excluded.

| Citation Information | April 2021, ACM Computing Surveys, Vol. 54, No. 3, Article 62. |
| --- | --- |
| Purpose (has the author formulated a problem?) | Review of Deep Learning based text classification |
| Subjects | Sentiment Analysis, Topic classification, question answering, deep learning |
| Methodology | Did not developed the SLR |
| Design and analysis | comprehensive review of more than 150 deep learning–based models for text classification developed in recent years, |
| Conclusions and results | Identified the gap in the latest research |
| Implications | Not applicable |
| Other (e.g. has relevant literature been evaluated? Contribution?) | Yes |

**Paper 26**

Deep Learning–based Text Classification: A Comprehensive Review

Excluded. Secondary Study

| Citation Information | August 2021. Technology in Society, Elsevier Journals |
| --- | --- |
| Purpose (has the author formulated a problem?) | Review of Challenges and Opportunities in opinion mining through social media |
| Subjects | Opinion Mining, Sentiment Analysis, Comprehensive Review |
| Methodology | Systematic Literature Review |
| Design and analysis | Formulation and Assessment of literature through three research questions |
| Conclusions and results | Outlined the potential challenges and opportunities in the latest research about the opinion mining through social media |
| Weaknesses | Sentiment Analysis as the core research area is not the topic in te study. |
| Strengths | Performed the SLR and provided all the relevant information about the process |
| Other (e.g. has relevant literature been evaluated? Contribution?) | Yes |

**Paper 27**

A review of social media-based public opinion analyses: Challenges and recommendations

Excluded. Secondary Study.

**Paper 28**

A Survey of Opinion Mining in Arabic: A Comprehensive System Perspective Covering Challenges and Advances in Tools, Resources, Models, Applications, and Visualizations

Excluded.

The string query extracted such papers due to the opinion mining keyword and therefore such papers are being excluded due to the defined research protocol.

| Citation Information | May 2019. ACM Trans. Asian Low-Resour. Lang. Inf. Process., Vol. 18, No. 3, Article 27 |
| --- | --- |
| Purpose (has the author formulated a problem?) | Survey of existing approaches available for opinion mining in Arabic Language |
| Subjects | Opinion Mining, Sentimental Analysis, Arabic Language |
| Methodology | The article aimed to provide a comprehensive literature survey for stateof-the-art advances in Arabic opinion mining. |
| Design and analysis | The survey outlined the advances in NLP software  tools, lexical sentiment and corpora resources, classification models, and applications of opinion mining. |
| Conclusions and results | While good progress and results have been achieved for Arabic sentiment analysis, specifically for MSA, a lot remains to be performed for dialectal Arabic. |
| Implications | None |
| Weaknesses | Not related to Urdu Sentiment Analysis |
| Strengths | SLR |
| Other (e.g. has relevant literature been evaluated? Contribution?) | YES |

| Citation Information | Aug 2018, Telematics and Informatics, Elsevier Journals |
| --- | --- |
| Purpose (has the author formulated a problem?) | The effect of lexicon-based approach in Supervised Machine Learning |
| Subjects | Machine learning, Sentiment Analysis, Urdu, Multidisciplinary, Urdu Sentiment Lexicon |
| Methodology | Testing these two different approaches for Urdu sentiment analysis |
| Design and analysis | Support Vector Machine, Decision Tree, K Nearest Neighbor, and Urdu Sentiment Analyzer are used in Lexicon-based approach |
| Conclusions and results | it is concluded that the Lexicon-based approach outperforms  Supervised Machine Learning approach not only in terms of Accuracy, Precision, Recall and F-measure but also in terms of economy of time and efforts used. |
| Implications | SML is more time consuming and costly than the Lexicon-based approach, for performing Urdu SA in multiple domains. |
| Other (e.g. has relevant literature been evaluated? Contribution?) | YES |

**Paper 29**

Lexicon-based approach outperforms Supervised Machine Learning approach for Urdu Sentiment Analysis in multiple domains

Included.

**Paper 30**

A Review on Sentiment Analysis Methodologies, Practices and Applications

| Citation Information | February 2020, International Journal of Scientific & Technology Research Volume 9, Issue 02 |
| --- | --- |
| Purpose (has the author formulated a problem?) | Review of recent trends in sentiment analysis |
| Subjects | Sentiment Analysis, Opinion Mining, Challenges, Machine Learning |
| Methodology | Comprehensive review of existing approaches |
| Design and analysis | Document level, sentence level and aspect level SA |
| Conclusions and results | In this paper, our examination represents machine learning procedures perform relatively better as compared to other computational models. |
| Implications | Could not found |
| Weaknesses | Indian Journal, Not Impact Factor, and so on |
| Strengths | Explained in detail, more like a blog post. |
| Other (e.g. has relevant literature been evaluated? Contribution?) | Yes. |

Included.

**Paper 31**

A survey on classification techniques for opinion mining and sentiment analysis

| Citation Information | Artif Intell Rev (2019) 52:1495–1545 |
| --- | --- |
| Purpose (has the author formulated a problem?) | Survey on classification methods for SA and Opinion Mining |
| Subjects | Sentiment Analysis, Opinion Mining, Machine Learning, |
| Methodology | Comprehensive survey for SA and opinion mining |
| Design and analysis | The study presents a complete, multilateral, and systematic review of opinion mining and sentiment analysis to classify available methods and compare their advantages and drawbacks |
| Conclusions and results | Identified the gap in the existing research about various aspects. |
| Implications | Could not find any. |
| Weaknesses | Old paper. Not specifically related to Urdu Sentiment Anlaysis |
| Strengths | Highlighted research gaps in detail with references to each area. |
| Other (e.g. has relevant literature been evaluated? Contribution?) | Yes |

Excluded. Not a Primary Study.

**Paper 32**

Scientific Text Sentiment Analysis using Machine Learning Techniques

| Citation Information | Dec 2019, (IJACSA) International Journal of Advanced Computer Science and Applications, Vol. 10, No. 12, 2019. |
| --- | --- |
| Purpose (has the author formulated a problem?) | Effect of using machine learning algorithm using scientific text for sentiment analysis |
| Subjects | Scientific text, sentiment analysis, machine learning models |
| Methodology | sentiment analysis of scientific articles using citation sentences is carried out using an existing constructed annotated corpus. accuracy of the system is evaluated using different evaluation metrics e.g. F-score and Accuracy score |
| Design and analysis | Naïve-Bayes (NB), Support Vector Machine (SVM), Logistic Regression (LR), Decision Tree (DT), K-Nearest Neighbor (KNN) and Random Forest (RF) |
| Conclusions and results | method achieved a maximum of about 9% improved results as compared to the base system. |
| Implications | Improved results |
| Weaknesses | Relatively fewer details are provided. |
| Strengths |  |
| Other (e.g., has relevant literature been evaluated? Contribution?) | Yes. |

Included.

**Paper 33**

Joint Sentiment/Topic Model for Sentiment Analysis

| Citation Information | Lin, C., & He, Y. (2009, November). Joint sentiment/topic model for sentiment analysis. In *Proceedings of the 18th ACM conference on Information and knowledge management* (pp. 375-384). |
| --- | --- |
| Purpose (has the author formulated a problem?) | Proposed and validated a novel model for Sentiment Analysis |
| Subjects | Sentiment Analysis, Topic modeling, topic mining, |
| Methodology | The study proposed a novel probabilistic modeling framework based on La-tent Dirichlet Allocation (LDA), called joint sentiment/topic model (JST) |
| Design and analysis | Unlike other machine learning approaches to sentiment classification which often require labeled corpora for classifier training, the proposed JST model is fully unsupervised |
| Conclusions and results | Preliminary experiments have shown promising results achieved by JST |
| Implications | Not listed |
| Weaknesses | The paper is outdated. |
| Strengths | Outlines the legacy research carried out in the previous decade. |
| Other (e.g. has relevant literature been evaluated? Contribution?) | Yes. |

Included. Legacy paper could be used in the literature survey for the review paper.

**Paper 34**

Aspect-based Opinion Mining from Product Reviews

| Citation Information | Marrese-Taylor, E., Velásquez, J. D., & Bravo-Marquez, F. (2014). A novel deterministic approach for aspect-based opinion mining in tourism products reviews. Expert Systems with Applications, 41(17), 7764-7775. |
| --- | --- |
| Purpose (has the author formulated a problem?) | mining online web-based reviews (opinion mining) |
| Subjects | Opinion Mining, Product Reviews, Aspect-based Sentiment Analysis |
| Methodology | Aspect based opinion mining using online product reviews |
| Design and analysis |  |
| Conclusions and results | The study outperformed the existing studies. |
| Implications | Improvement in results due to proposed technique. |
| Weaknesses | Not remotely related to Urdu Sentiment Analysis |

Excluded. Not relevant.

**Paper 35**

An Experimental Analysis of Clustering Sentiments for Opinion Mining

| Citation Information | Babu, A. G., Kumari, S. S., & Kamakshaiah, K. (2017, January). An experimental analysis of clustering sentiments for opinion mining. In Proceedings of the 2017 International Conference on Machine Learning and Soft Computing (pp. 53-57). |
| --- | --- |
| Purpose (has the author formulated a problem?) | Use of Clustering-based learning for opinion mining |
| Subjects | Opinion mining, unsupervised learning, sentiment analysis |
| Methodology | K-means clustering technique applied on a sample twitter dataset to cluster different sentiments in context with different features |
| Design and analysis | Training and validation of the system using the various machine learning tools |
| Implications | Improved process of opinion mining |
| Weaknesses | The work can be enhanced focusing on heterogeneous data such as images, videos for opinion mining |
| Strengths | Could not find. |
| Other (e.g. has relevant literature been evaluated? Contribution?) | Yes. |

Included. Shall be used in the literature survey.

**Paper 36**

Coupling Topic Modelling in Opinion Mining for Social Media Analysis

| Citation Information | Zhou, X., Tao, X., Rahman, M. M., & Zhang, J. (2017, August). Coupling topic modelling in opinion mining for social media analysis. In *Proceedings of the International Conference on Web Intelligence* (pp. 533-540). |
| --- | --- |
| Purpose (has the author formulated a problem?) | Opinion Mining using Social Media Analysis |
| Subjects | Opinion Mining, Sentiment Analysis, Social Media Analysis |
| Methodology | proposed a new method that combines the opining mining and context-based topic modelling to analyze public opinions on social media data. |
| Design and analysis | Context based topic modelling is used to categorize data in groups and discover hidden communities in data group. The unwanted data group  discovered by the topic model then will be discarded. |
| Conclusions and results | experimental results demonstrate that, with the help of topic modelling, our social media analysis model is accurate and effective. |
| Implications | Not listed. |
| Weaknesses | No comparative analysis with existing/baseline methods. |
| Strengths | Could not find any. |
| Other (e.g. has relevant literature been evaluated? Contribution?) | Yes. |

Included. The paper would be included in the different techniques for opinion mining and classification section of our study.

**Paper 37**

Urdu Language Based Information Dissemination System for Low-Literate Farmers

| Citation Information | Idrees, F., Qadir, J., Mehmood, H., Hassan, S. U., & Batool, A. (2019, January). Urdu language based information dissemination system for low-literate farmers. In Proceedings of the Tenth International Conference on Information and Communication Technologies and Development (pp. 1-5). |
| --- | --- |
| Purpose (has the author formulated a problem?) | Assisting low-literate farmers using Urdu based information dissemination system |
| Subjects | Information Dissemination System, Urdu language, |
| Methodology | Feed back base web development system |
| Design and analysis | Requirement gathering, design, feedback, improvement |
| Conclusions and results | Development of stand-alone platform |
| Implications | Not listed. |
| Weaknesses | Not related to our problem. |
| Strengths | Could not find. |
| Other (e.g. has relevant literature been evaluated? Contribution?) | Not relevant. |

Excluded. Not relevant.

**Paper 38**

Roman Urdu Reviews Dataset for Aspect Based Opinion Mining

| Citation Information | Zahid, R., Idrees, M. O., Mujtaba, H., & Beg, M. O. (2020, September). Roman urdu reviews dataset for aspect based opinion mining. In 2020 35th IEEE/ACM International Conference on Automated Software Engineering Workshops (ASEW) (pp. 138-143). IEEE. |
| --- | --- |
| Purpose (has the author formulated a problem?) | development of novel dataset for aspect base opinion mining |
| Subjects | Roman Urdu, dataset development, aspect-based analysis, opinion mining |
| Methodology | Presents a proposed reviews dataset (a RU dataset) of mobile reviews that has been manually annotated with multi-aspect sentiment labels  at the sentence-level |
| Design and analysis | It presents base-line results using different Machine Learning (ML) algorithms |
| Conclusions and results | results demonstrate 71% F1-score for aspect detection and 64% for aspect-based polarity. |
| Implications | Addition into existing limited Urdu language corpora. |
| Weaknesses | Dataset not available publicly, |
| Strengths | Could not found. |
| Other (e.g. has relevant literature been evaluated? Contribution?) | yes |

Included. Would be included in the related work section of the paper.

**Paper 39**

Learning sentiment from students' feedback for real-time interventions in classrooms

| Citation Information | Altrabsheh, N., Cocea, M., & Fallahkhair, S. (2014, September). Learning sentiment from students’ feedback for real-time interventions in classrooms. In International conference on adaptive and intelligent systems (pp. 40-49). Springer, Cham. |
| --- | --- |
| Purpose (has the author formulated a problem?) | Analysis of the feedback from student by performing sentiment analysis |
| Subjects | Student feedback analysis, sentiment analysis, machine learning method |
| Methodology | Naive Bayes, Complement Naive Bayes (CNB), Maximum Entropy and Support Vector Machine (SVM) were trained using real students' feedback. |
| Design and analysis | Explored several methods that could be used for learning sentiment from student’s feedback. |
| Conclusions and results | SVM resulting in the highest accuracy at 94%, followed by CNB at 84%. |
| Implications | The technique improved the baseline results |
| Weaknesses | Could not find. |
| Strengths | Could not find. |
| Other (e.g. has relevant literature been evaluated? Contribution?) | Yes |

Included. The paper would be included to discuss various application of sentiment analysis in modern day sciences.

**Paper 40**

Sentiment Analysis Based on Reviews Using Machine Learning Techniques

| Citation Information | Sattar, A., & Fatima, J. (2021). Sentiment Analysis Based on Reviews Using Machine Learning Techniques. Pakistan Journal of Engineering and Technology, 4(2), 149-152. |
| --- | --- |
| Purpose (has the author formulated a problem?) | Using social media reviews for sentiment analysis using machine learning methods. |
| Subjects | Sentiment Analysis, Reviews dataset, Machine learning methods |
| Methodology | Roman Urdu reviews sentiment analysis using various machine learning methods |
| Conclusions and results | SVM performs the best result on used data sets |
| Implications | accuracy against dataset-1 that is 69%, dataset-2 is 73% and dataset-3 is 76% |
| Weaknesses | Not listed. |
| Strengths | The accuracy in the dataset as well as to measure the precision, recall, and F-measure score of data on the different classifiers |
| Other (e.g. has relevant literature been evaluated? Contribution?) | Yes. |

Included. The same paper can be replicated on Urdu language corpora.

**Paper 41**

A Sentiment Analysis System to Improve Teaching and Learning

| Citation Information | Rani, S., & Kumar, P. (2017). A sentiment analysis system to improve teaching and learning. Computer, 50(5), 36-43. |
| --- | --- |
| Purpose (has the author formulated a problem?) | Effect of sentiment analysis in teaching and learning |
| Subjects | Education, sentiment analysis, text classification, |
| Methodology | It is a book chapter that highlights the effect of incorporating sentiment analysis-based system for improving education and learning process. |
| Other (e.g., has relevant literature been evaluated? Contribution?) | No. |

included. It is a book chapter that can be used to cite different points in introduction.

**Paper 42**

Discriminative Feature Spamming Technique for Roman Urdu Sentiment Analysis

| Citation Information | Mehmood, K., Essam, D., Shafi, K., & Malik, M. K. (2019). Discriminative feature spamming technique for roman urdu sentiment analysis. IEEE Access, 7, 47991-48002. |
| --- | --- |
| Purpose (has the author formulated a problem?) | Sentiment analysis of Roman Urdu text |
| Subjects | Spamming Technique, Sentiment Analysis, Roman Urdu, Descriptive |
| Methodology | novel term weighting technique, discriminative feature spamming  technique (DFST), |
| Design and analysis | The DFST identifies distinctive terms, based on a term utility criterion (TUC), and then spams them to increase their discriminative power |
| Conclusions and results | The experimental results show that the DFST outperformed a  set of time-tested term weighting schemes, from the information retrieval field. |
| Weaknesses | The study used roman Urdu for experiments. We can use Urdu corpora for doing the same. |
| Strengths | A cross-scheme comparison was performed, which showed that the results obtained by using the newly proposed DFST, were statistically significant and better than previous approaches. |
| Other (e.g. has relevant literature been evaluated? Contribution?) | Yes. |

Included.

Paper 43

Roman-Urdu News Headline Classification with IR Models using Machine Learning Algorithms

| Citation Information | Hassan, S. M., Ali, F., Wasi, S., Javeed, S., Hussain, I., & Ashraf, S. N. (2019). Roman-Urdu News Headline Classification with IR Models using Machine Learning Algorithms. Indian Journal of Science and Technology, 12(35), 1-9. |
| --- | --- |
| Purpose (has the author formulated a problem?) | Headline Classification |
| Subjects | Sentiment Analysis, Sentiment Classification, IR Models, Machine Learning Algorithms |
| Methodology | Development of novel headline corpus, TF-IDF and Count Vector for feature extraction |
| Design and analysis | corpus contains 12319 news headlines which contain seven categories i.e., Accident, Sports, Weather, Arrest, Conference, Operation and Violence |
| Conclusions and results | model predicts best result to identify desire class on SGD classifier which gives 93.50% accuracy. |
| Implications | Improved Headline classification |
| Weaknesses | Not relevant. |
| Strengths | It is recommended that SGD Classifiers should be used in roman-Urdu news headline text classification. |
| Other (e.g. has relevant literature been evaluated? Contribution?) | Yes. |

Included. The paper would be used to list the applications of sentiment analysis in different research areas.

**Paper 44**

Urdu Sentiment Analysis

| Citation Information | (IJACSA) International Journal of Advanced Computer Science and Applications, Vol. 9, No. 9, 2018 |
| --- | --- |
| Purpose (has the author formulated a problem?) | Sentiment Analysis, Urdu language, Survey |
| Weaknesses | Survey paper |
| Strengths | Could not find any. |
| Other (e.g. has relevant literature been evaluated? Contribution?) | Did not checked. |

Excluded. Survey Paper

**Paper 45**

Sentiment Analysis of User Reviews about Hotel in Roman Urdu

| Citation Information | Nazir, M. K., Ahmad, M., Ahmad, H., Qayum, M. A., Shahid, M., & Habib, M. A. (2020, December). Sentiment Analysis of User Reviews about Hotel in Roman Urdu. In 2020 14th International Conference on Open Source Systems and Technologies (ICOSST) (pp. 1-5). IEEE. |
| --- | --- |
| Purpose (has the author formulated a problem?) | Roman Urdu based Sentiment Analysis From text of Hostel Reviews |
| Subjects | Sentiment Analysis, Roman Urdu, User Reviews, |
| Methodology | Machine learning based models are trained using the developed corpus |
| Design and analysis | The authors developed a novel dataset of 3000 reviews in roman urdu, for testing the proposed hypothesis. |
| Conclusions and results | The study reveals that logistical regression and SVM outperformed the other ML assisted model. |
| Implications | Improved sentiment analysis of reviews from hotel |
| Weaknesses | While It’s a conference paper, however, still can be used in the applications section of our study. |
| Strengths | Could not find any. |
| Other (e.g. has relevant literature been evaluated? Contribution?) | Yes. |

Excluded. Conference paper, results are not given in the paper and only the proposed idea is listed.

**Paper 46**

Sentiment Analysis for Urdu News Tweets Using Decision Tree

| Citation Information | Bibi, R., Qamar, U., Ansar, M., & Shaheen, A. (2019, May). Sentiment analysis for urdu news tweets using decision tree. In 2019 IEEE 17th International Conference on Software Engineering Research, Management and Applications (SERA) (pp. 66-70). IEEE. |
| --- | --- |
| Purpose (has the author formulated a problem?) | Effect of using decision trees in the Urdu news classification using sentiment analysis |
| Subjects | Sentiment Analysis, Urdu news, decision trees |
| Methodology | After preprocessing, the study performs the step feature vector. The decision tree algorithm is used afterwards to achieve the results. |
| Conclusions and results | 90.00 % accuracy of the proposed methodology. The achieve accuracy is encouraging as compare to previous sentiment on Urdu using machine learning. |
| Weaknesses | Low size of the used dataset with only 500 tweets. |
| Strengths | Could not find any. |
| Other (e.g. has relevant literature been evaluated? Contribution?) | Yes. |

Included. The idea can be explored to carry out new research from that point.

**Paper 47**

Sentiment mining in a collaborative learning environment: capitalising on big data

| Citation Information | Jena, R. K. (2019). Sentiment mining in a collaborative learning environment: capitalising on big data. Behaviour & Information Technology, 38(9), 986-1001. |
| --- | --- |
| Purpose (has the author formulated a problem?) | The use of sentiment learning to improve the learning environments. |
| Subjects | Educational Environment, Sentiment Analysis, Bid data |
| Methodology | The developed SM techniques using big data frameworks can be scaled and made adaptable for source variation, velocity and veracity to maximize value mining for the benefit of students, faculties and other stakeholders. |
| Conclusions and results | the machine learning technique CNB achieved the best performance with an average accuracy of 92%. |
| Implications | modelling and predicting students’ emotions (Amused, Anxiety, Bored,  Confused, Enthused, Excited, Frustrated, etc.) using the big data frameworks |
| Weaknesses | Could not find. |
| Strengths | Improved learning process. |
| Other (e.g. has relevant literature been evaluated? Contribution?) | Yes. |

Included. The paper provides a comprehensive overview of the techniques that are explored in educational sentiment analysis.

**Paper 48**

Effective use of evaluation measures for the validation of best classifier in Urdu sentiment analysis

| Citation Information | Mukhtar, N., Khan, M. A., & Chiragh, N. (2017). Effective use of evaluation measures for the validation of best classifier in Urdu sentiment analysis. Cognitive Computation, 9(4), 446-456. |
| --- | --- |
| Purpose (has the author formulated a problem?) | Identification of best classifier for Urdu sentiment analysis |
| Subjects | Sentiment Analysis, Machine Learning, Validation methods |
| Methodology | PART, Navies Bayes multinomial Text, Lib SVM (support vector machine), decision tree (J48), and k nearest neighbor (KNN, IBK) are employed using Weka. |
| Design and analysis | Three different validation parameters are considered for performing validation of sentiment analysis. |
| Conclusions and results | after using 10-fold cross-validation, three topmost classifiers, i.e., Lib SVM, J48, and IBK are selected because of high accuracy, precision, recall, and F-measure. |
| Weaknesses | Could not find any. |
| Strengths | Improved results |
| Other (e.g., has relevant literature been evaluated? Contribution?) | Yes. |

Included. The paper provides a methodology that improves the results on the baseline dataset.

**Paper 49**

The essential of sentiment analysis and opinion mining in social media: Introduction and survey of the recent approaches and techniques

| Citation Information | Soong, H. C., Jalil, N. B. A., Ayyasamy, R. K., & Akbar, R. (2019, April). The essential of sentiment analysis and opinion mining in social media: Introduction and survey of the recent approaches and techniques. In 2019 IEEE 9th symposium on computer applications & industrial electronics (ISCAIE) (pp. 272-277). IEEE. |
| --- | --- |
| Purpose (has the author formulated a problem?) | Survey of the approaches and challenges in the field of opinion mining and sentiment analysis |
| Subjects | Sentiment Analysis, Opinion Mining, social media |
| Methodology | Detailed SLR regarding emotion mining, opinion mining, and sentiment analysis methods |
| Design and analysis | Machine learning based approaches, and lexicon analysis-based approaches. |
| Conclusions and results | to overcome the weaknesses of both supervised and unsupervised method, semi-supervised or hybrid method is introduced to gain the strength of both supervised and unsupervised method. |
| Other (e.g. has relevant literature been evaluated? Contribution?) | Yes. |

Included. The paper can has been used to get other paper from snowballing phenomenon.

**Paper 50**

Lexicon-based sentiment analysis in Persian

| Citation Information | Basiri, M. E., Ghasem-Aghaee, N., & Reza, A. (2017). Lexicon-based sentiment analysis in Persian. Current and Future Developments in Artificial Intelligence, 30, 154-183. |
| --- | --- |
| Purpose (has the author formulated a problem?) | The impact of lexicon-based sentiment analysis in Persian language. |
| Subjects | Sentiment Analysis, Opinion Mining, Lexicon based approaches and methods |
| Methodology | Developed a lexicon for the sentiment analysis of Persian language. The lexicon associates the sentiment words with the sentiment strengths in lexicon. |
| Design and analysis | Word spelling, stemming, word spacing as other such issues have been targeted in the proposed research methodology. |
| Conclusions and results | The approaches outperformed the existing supervised learning-based approaches on the baseline dataset. |
| Implications | The improved results indicate the accuracy of the proposed methodology. |
| Weaknesses | Not related to Urdu Sentiment Analysis |
| Strengths | Could not find. |
| Other (e.g. has relevant literature been evaluated? Contribution?) | Yes |

Excluded. Such papers with similar methodology on the Persian language have been discussed in the earlier section of the documents.

**Paper 51**

Lexicon-based sentiment analysis for Urdu language

| Citation Information | Rehman, Z. U., & Baja, I. S. (2016, August). Lexicon-based sentiment analysis for Urdu language. In 2016 sixth international conference on innovative computing technology (INTECH) (pp. 497-501). IEEE. |
| --- | --- |
| Purpose (has the author formulated a problem?) | The impact of the lexicon-based sentiment analysis using Urdu language corpora. |
| Subjects | Sentiment Analysis, Urdu language, Lexicon based approach, social media |
| Design and analysis | The paper aims at creating an application for sentiment analysis of Urdu comments on various websites. |
| Conclusions and results | The F-measure achieved by this proposed system is 0.73, results of 66% accuracy. |
| Implications | Improved results |
| Other (e.g. has relevant literature been evaluated? Contribution?) | Yes. |

Included. Relevant paper

**Paper 52**

Enhancing aspect-based sentiment analysis of Arabic hotels' reviews using morphological, syntactic and semantic features

| Citation Information | Al-Smadi, M., Al-Ayyoub, M., Jararweh, Y., & Qawasmeh, O. (2019). Enhancing aspect-based sentiment analysis of Arabic hotels’ reviews using morphological, syntactic, and semantic features. Information Processing & Management, 56(2), 308-319. |
| --- | --- |
| Purpose (has the author formulated a problem?) | The use of machine learning approaches for the sentiment analysis of hotel reviews in Arabic language |
| Subjects | Sentiment Analysis, aspect-based approach, Arabic language, Machine Learning methods |
| Methodology | Aspect-Based Sentiment Analysis (ABSA) of Hotels’ Arabic reviews using supervised machine learning |
| Design and analysis | employs state-of-the-art research of training a set of classifiers with morphological, syntactic, and semantic features to address the research tasks |
| Conclusions and results | evaluation results show that all classifiers in the proposed approach outperform the baseline approach, and the overall enhancement for the best performing classifier (SVM) is around 53% for T1, around 59% for T2, and around 19% in T3. |
| Weaknesses | Could not find. |
| Strengths | Impact factor recognized paper with robust methodology. |
| Other (e.g., has relevant literature been evaluated? Contribution?) | Yes. |

Included. Same could be applied for sentiment analysis using Urdu language.

**Paper 53**

Sentiment classification of Roman-Urdu opinions using Naïve Bayesian, Decision Tree and KNN classification techniques

| Citation Information | Bilal, M., Israr, H., Shahid, M., & Khan, A. (2016). Sentiment classification of Roman-Urdu opinions using Naïve Bayesian, Decision Tree and KNN classification techniques. Journal of King Saud University-Computer and Information Sciences, 28(3), 330-344. |
| --- | --- |
| Purpose (has the author formulated a problem?) | Sentiment classification of Roman-Urdu opinions using machine learning classifiers |
| Subjects | Sentiment analysis, roman Urdu, Supervised learning, Machine Learning |
| Methodology | Naïve Bayesian, Decision Tree and KNN were used to train and validate on the developed corpus. |
| Conclusions and results | The results show that Naïve Bayesian outperformed Decision Tree and KNN in terms of more accuracy, precision, recall and F-measure. |
| Implications | Machine learning models performed better on Roman Urdu dataset. |
| Weaknesses | Comparatively limited dataset |
| Strengths | Could not identify. |
| Other (e.g. has relevant literature been evaluated? Contribution?) | Yes. |

Included. Relevant Paper.

**Paper 54**

Sentiment Analysis on Urdu Tweets Using Markov Chains

| Citation Information | Nasim, Z., & Ghani, S. (2020). Sentiment Analysis on Urdu Tweets Using Markov Chains. SN Computer Science, 1(5), 1-13. |
| --- | --- |
| Purpose (has the author formulated a problem?) | Sentiment prediction of Urdu tweets |
| Subjects | Sentiment Analysis, Urdu language, Social Media, Twitter, Markov Chains |
| Methodology | work focuses on developing a 3-class (positive, negative, and neutral) sentiment classification model for the Urdu language. |
| Design and analysis | The experiments were conducted on the labeled corpus of Urdu tweets extracted from the Twitter network. |
| Conclusions and results | the results showed that the proposed approach outperforms the lexicon-based and traditional machine learning-based approaches of sentiment analysis. |
| Implications | Improved results |
| Weaknesses | Could not find. |
| Strengths | To the best of our knowledge, there is no such corpus available publicly in the Urdu Language. The labeled dataset is available on GitHub (<https://github.com/zarmeen92/urdutweets>) |
| Other (e.g. has relevant literature been evaluated? Contribution?) | Yes. |

Included and selected for critical read.

**Paper 55**

A framework for Arabic sentiment analysis using supervised classification

| Citation Information | Duwairi, R. M., & Qarqaz, I. (2016). A framework for Arabic sentiment analysis using supervised classification. International Journal of Data Mining, Modelling and Management, 8(4), 369-381. |
| --- | --- |
| Purpose (has the author formulated a problem?) | Development of a novel framework for the Arabic sentiment analysis using supervised learning methods. |
| Subjects | Sentiment Analysis, Arabic Language, Supervised Learning, Classification |
| Methodology | Three classifiers on an in-house developed dataset of tweets/comments including the Naïve Bayes, SVM and K-nearest neighbour classifiers were employed. |
| Design and analysis | The binary model, term frequency and term frequency inverse document frequency were used to assign weights to the tokens of tweets/comments. |
| Conclusions and results | The highest accuracy was equal to 69.97 was achieved by the NB classifier when TF was used. |
| Implications | The results show that alternating between the three weighting schemes slightly affects the accuracies. |
| Weaknesses | Not related to Urdu language-based Sentiment Analysis |
| Strengths | Could not identify. |
| Other (e.g. has relevant literature been evaluated? Contribution?) | Yes. |

Included. Relevant paper. The approach/methodology is same as used in the Urdu language sentiment analysis.

**Paper 56**

Lexicon based sentiment analysis of Urdu text using SentiUnits

| Citation Information | Syed, A. Z., Aslam, M., & Martinez-Enriquez, A. M. (2018, November). Lexicon based sentiment analysis of Urdu text using SentiUnits. Springer, Berlin, Heidelberg. |
| --- | --- |
| Purpose (has the author formulated a problem?) | Impact of using SentiUnits for lexicon-based sentiment analysis of Urdu language |
| Subjects | Sentiment Analysis, Urdu language, Lexicon based approach, |
| Methodology | The approach towards sentiment analysis is based on the identification and extraction of SentiUnits from the given text, using shallow parsing. Development of lexicon for this poor resource language. |
| Design and analysis | The goal is to highlight the linguistic (grammar and morphology) as well as technical aspects of this multidimensional research problem. |
| Conclusions and results | The SVM outperformed the state-of-the-art baseline models by 15% accuracy. |
| Implications | Improved results as compared to previous studies. |
| Weaknesses | could not identify. |
| Other (e.g. has relevant literature been evaluated? Contribution?) | Yes. |

Included. Relevant paper. Shall be included in the literature survey section of the paper.

**Paper 57**

Roman Urdu sentiment analysis using Machine Learning with best parameters and comparative study of Machine Learning algorithms

| Citation Information | Aziz, S., Ullah, S., Mughal, B., Mushtaq, F., & Zahra, S. (2020). Roman Urdu sentiment analysis using Machine Learning with best parameters and comparative study of Machine Learning algorithms. Pakistan Journal of Engineering and Technology, 3(2), 172-177. |
| --- | --- |
| Purpose (has the author formulated a problem?) | To propose a technique for roman Urdu sentiment analysis and conduct the comparative analysis of existing methodologies |
| Subjects | Machine learning, Sentiment Analysis, Comparative Analysis, Roman Urdu language |
| Methodology | We have collected the Dataset from Kaggle containing 21000  values with manually annotated and prepare the data for machine learning |
| Design and analysis | we apply different machine learning algorithms (SVM, Logistic regression, Random Forest, Naïve Bayes, AdaBoost, KNN) (Bowers et al. 2018) with different parameters and kernels and with TFIDF (Unigram, Bigram, Uni-Bigram) |
| Conclusions and results | Robust linear Regression model delivered us the best estimators with 82.19%. |
| Implications | Performed robust sentiment analysis using newly developed dataset |
| Weaknesses | Could not identify yet. |
| Other (e.g. has relevant literature been evaluated? Contribution?) | Yes. |

Included. Relevant Paper.

**Paper 58**

Sentiment Analysis of Roman-Urdu Tweets about Covid-19 Using Machine Learning Approach: A Systematic Literature

| Citation Information | Shah, S. M. W., Nadeem, M., & Mehboob, M. (2021). Sentiment Analysis of Roman-Urdu Tweets about Covid-19 Using Machine Learning Approach: A Systematic Literature. International Journal, 10(2). |
| --- | --- |
| Purpose (has the author formulated a problem?) | Sentiment Analysis of roman Urdu based tweets about covid-19 |
| Subjects | Sentiment analysis, covid-19, systematic literature review, machine learning methods |
| Methodology | Systematic literature review for covid-19 using machine learning approaches |
| Design and analysis | Identified research questions and performed the SLR. |
| Conclusions and results | Identified the gaps and challenges in the recent trends. |
| Implications | During the study we have observed that TF-IDF with Unigram and Bigram are the most selected features for the sentiment analysis of Roman –Urdu text. |
| Strengths | Could not identify. |
| Other (e.g., has relevant literature been evaluated? Contribution?) | Yes. |

Excluded. It is not a primary study.

**Paper 59**

Sentiment analysis of comment texts based on BiLSTM

| Citation Information | Xu, G., Meng, Y., Qiu, X., Yu, Z., & Wu, X. (2019). Sentiment analysis of comment texts based on BiLSTM. IEEE Access, 7, 51522-51532. |
| --- | --- |
| Purpose (has the author formulated a problem?) | Using BiLSTM for sentiment analysis of comment texts |
| Subjects | Sentiment analysis, BiLSTM, comment text |
| Methodology | an improved word representation method is proposed, which integrates the contribution of sentiment information into the traditional TF-IDF algorithm and generates weighted word vectors. |
| Design and analysis | The weighted word vectors are input into bidirectional long short term memory (BiLSTM) to capture the context information effectively, and the comment vectors are better represented. |
| Conclusions and results | The experimental results show that the proposed sentiment analysis method has higher precision, recall, and F1 score. |
| Implications | Improved results. |
| Weaknesses | the sentiment analysis method of comments based on BiLSTM consumes a long time in the training model. |
| Strengths | Could not identify. |
| Other (e.g., has relevant literature been evaluated? Contribution?) | Yes. |

Included. Also selected for critical thinking.

**Paper 60**

Sentence level sentiment analysis using Urdu nouns

| Citation Information | Hashim, F., & Khan, M. (2016). Sentence level sentiment analysis using urdu nouns. Department of Computer Science, University of Peshawar, Pakistan, 101-108. |
| --- | --- |
| Purpose (has the author formulated a problem?) | Urdu nouns for sentence level sentiment analysis |
| Subjects | Sentiment analysis, sentiment analyzer, sentence level approach, Urdu noun |
| Methodology | Sentiment analyzer is proposed along with a lexicon-based approach. |
| Design and analysis | Corpus development is also carried out. |
| Conclusions and results | Outperformed the existing machine learning base models. |
| Implications | Improvement in results. |
| Other (e.g. has relevant literature been evaluated? Contribution?) | Yes. |

Included. Relevant Papers.
